# Dynamics of laser-induced cavitation bubble during expansion over sharp-edge geometry submerged in liquid – an inside view by diffuse illumination

**DOI:** 10.1016/j.ultsonch.2021.105460

**Published:** 2021-01-09

**Authors:** Matej Senegačnik, Kohei Kunimoto, Satoshi Yamaguchi, Koki Kimura, Tetsuo Sakka, Peter Gregorčič

**Affiliations:** a Faculty of Mechanical Engineering, University of Ljubljana, Aškerčeva 6, 1000 Ljubljana, Slovenia; b Department of Energy and Hydrocarbon Chemistry, Kyoto University, Nishikyo, Kyoto 615-8510, Japan

**Keywords:** PEG, polyethylene glycol, Laser ablation in liquids, Cavitation bubble, Illumination, Shadowgraphy, Nanoparticle production, Re-entrant jet

## Abstract

Laser ablation in liquids is growing in popularity for various applications including nanoparticle production, breakdown spectroscopy, and surface functionalization. When laser pulse ablates the solid target submerged in liquid, a cavitation bubble develops. In case of “finite” geometries of ablated solids, liquid dynamical phenomena can occur inside the bubble when the bubble overflows the surface edge. To observe this dynamics, we use diffuse illumination of a flashlamp in combination with a high-speed videography by exposure times down to 250  ns. The developed theoretical modelling and its comparison with the experimental observations clearly prove that this approach widens the observable area inside the bubble. We thereby use it to study the dynamics of laser-induced cavitation bubble during its expansion over a sharp-edge (“cliff-like” 90°) geometry submerged in water, ethanol, and polyethylene glycol 300. The samples are 17  mm wide stainless steel plates with thickness in the range of 0.025–2  mm. Bubbles are induced on the samples by 1064-nm laser pulses with pulse durations of 7–60  ns and pulse energies of 10–55  mJ. We observe formation of a fixed-type secondary cavity behind the edge where low-pressure area develops due to bubble-driven flow of the liquid. This occurs when the velocity of liquid overflow exceeds ~20  m s^−1^. A re-entrant liquid injection with up to ~40  m s^−1^ velocity may occur inside the bubble when the bubble overflows the edge of the sample. Formation and characteristics of the jet evidently depend on the relation between the breakdown-edge offset and the bubble energy, as well as the properties of the surrounding liquid. Higher viscosity of the liquid prevents the generation of the jet.

## Highlights

• Diffuse illumination enables fluid dynamics observation inside cavitation bubbles.
• Dynamics of laser-induced bubbles near a 90° sharp solid-liquid boundary is studied.
• Bubble-driven overflow of cliff-like solid edge forms a fixed-type secondary cavity.
• Re-entrant injection of surrounding liquid into the bubble is detected at the edge.
• Characteristics of re-entrant jet depend on liquid viscosity and surface tension.

## Graphical abstract

**Figure:**
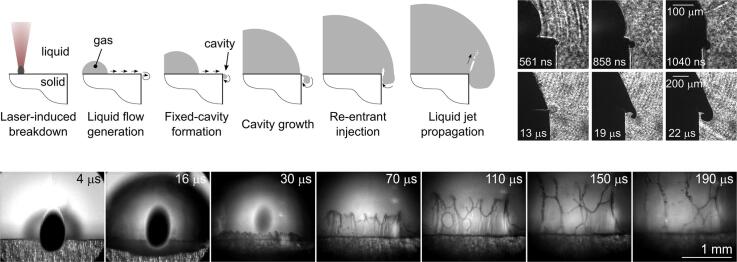

## 1 Introduction

The field of laser ablation in liquids (LAL) has exhibited significant development since the beginning of the 21st century due to its implementation in a broad range of applications, including laser-induced breakdown spectroscopy [1], [2], [3], surface functionalization [4], [5], [6], [7], and nanoparticle production [8], [9], [10], [11]. Although numerous studies have been devoted to clarification of the laser-induced cavitation bubble dynamics, some aspects of bubble development and nanoparticle formation still remain poorly understood. In this context, some authors suggest that the nanoparticles are generated in the liquid environment outside of the bubble [12], while others propose that they form inside the bubble during its expansion phase [13].

Most of the studies consider the dynamics of bubbles that are induced in infinite [14], [15] and semi-infinite liquid environments (i.e., containing a large flat liquid/solid [16], [17] or liquid/liquid [18], [19] interface). In case of laser-induced breakdown near a solid (rigid) boundary that is immersed into liquid, the cavitation bubble collapses asymmetrically and multiple reports of liquid injections inside the cavitation bubble can be found in the literature [16], [17], [18], [20], [21], [22], [23]. Extensive studies of this phenomenon were mainly performed to improve the understanding of the prevalent mechanism that is responsible for damage caused by cavitation [24], [25]. Bubbles in these studies were thereby induced in close proximity of an “infinitely” large flat rigid surface. In this case, a liquid jet with “tip” velocity in the order of 100 m s^−1^ develops towards the solid surface during the collapse phase. The formation of the jet is generally explained by reduced bubble wall velocity adjacent to the rigid surface [20]. Recent studies also show that the evolution of the cavitation bubble and its collapse can be additionally influenced by the geometry of the solid surrounding the breakdown [26], [27]. Specifically, it was demonstrated that ablation of unconventional deformable geometry, such as a thin wire [28], results in a “spring board” effect [29], so that the collapse of the laser-induced cavitation bubble takes place away from the irradiated surface [30]. In this case, an increased nanoparticle productivity was reported [31] and explained by the decreased redeposition of laser-generated nanoparticles to the donor surface during the bubble collapse.

One of the most common and straightforward methods used to study cavitation bubbles in the aforementioned studies is shadowgraphy [14], [18], [32], [33], [34], [35], [36]. Here, the main idea is to illuminate the bubble from the back and thereby cast its shadow onto an imaging sensor, which is usually in form of an industrial or high speed camera. Since the bubble wall is essentially a liquid-gas interface that separates two phases with different optical densities, the direction of illuminating light is altered upon crossing this interface. This effect is conveniently utilized by imaging techniques including schlieren photography [17], [37], [38] and laser-beam deflection [39], [40], [41], [42] or transmission [36], [43] probes, which acquire the signal based on the refractive index gradient. These methods enable fairly uncomplicated tracking of the overall bubble dynamics (i.e., observation of the movement and shape of its wall). However, for capturing the liquid dynamics *inside* the cavitation bubble (e.g., liquid injections and particle movement), different optical densities at the liquid-gas interface forming the bubble wall prove undesirable as significant amount of illuminating light incident on the bubble wall is deviated out of the objective’s aperture. This can be overcome by using X-ray illumination, since the real part of the refractive index of liquid and vapor is not drastically different for this part of the light spectrum. Thus, time resolved X-ray techniques including radiography [44] and small-angle X-ray scattering [13], [45] have been implemented in the studies of laser nanoparticle formation in liquids due to their good selectivity for condensed phases and high atomic weight materials [46]. This is specifically suitable for determining the presence, location, and size of noble metal nanoparticles. On the other hand, the disadvantage of these methods lies in the relatively weak signal during the measurements. Longer exposure times and/or averaging of many repetitions, therefore, need to be implemented, resulting in decreased temporal and spatial resolution of the acquired images.

The first aim of this paper is to show that a diffuse light source in the visible spectrum can be used to cope with the reflection and refraction of the illuminating light at the bubble wall. Our results prove that a diffuse light source increases the amount of light that is “transmitted” through the bubble. This widens the observable area inside the bubble and enables the tracking of *internal* liquid dynamics. For better understanding of the bubble imaging with visual light illumination, the first part of this paper is dedicated to a theoretical and experimental comparison of different illuminating conditions and their effects on perception of the cavitation bubbles.

Several studies have already demonstrated that using (unconventional) thin samples with 100  μm –1  mm thickness [27], [28], [29], [30], [31] for ablation can prove beneficial to nanoparticle production by altering the bubble oscillation dynamics. In this case, if the maximum diameter of the laser-induced cavitation bubble is larger than the size of the irradiated solid surface (bubbles induced by laser pulses with pulse energy in the range of several mJ are often of millimeter-size [14]), bubble overflow of the sample is inevitable. Thus, the second aim of this study is to gain a more detailed insight into dynamics following such “enwrapping” of a solid by a cavitation bubble. In the second part of this paper, we focus on phenomena that occur when the bubble expands over a sharp rigid edge. Instead of irradiating a conventional “infinitely” large flat solid surface, the irradiated samples exhibit a “cliff-like” 90° edge distanced 12.5  μm −1.7  mm away from the position of optical breakdown. Subsequent dynamics is observed from different sides. With implementation of appropriate diffuse illumination, the liquid dynamics *inside* the bubbles is monitored and evaluated. As the laser-induced cavitation bubble expands over the sharp edge of the solid surface, re-entrant injection of liquid into the bubble is captured by using high-speed videography. Our results also reveal a secondary cavity that is developed behind the sharp edge due to low pressure area formed as a result of bubble-driven flow of the surrounding liquid. To clarify the effects of liquid properties on formation and characteristics of the jets and cavities, the experiments are performed in water, ethanol, and polyethylene glycol 300.

## 2 Experimental

### 2.1 Materials and liquids

The cavitation bubble was induced by focusing a nanosecond laser pulse on the top face of a stainless-steel (SS) sample in close proximity to a “cliff-like” 90° solid edge (see Fig. S1). Dimensions of the samples equaled ~17 × 6  mm^2^ (width×height) with thicknesses ranging from 25  μm to 2  mm. During irradiation, samples were submerged into water, ethanol, or polyethylene glycol 300 (PEG) approximately 10  mm below the liquid surface. The most relevant physical properties of the liquids used in the experiments are presented in Table 1.

**Table 1:** Properties of liquids (at 20 °C) used in the experiments.

Liquid	Density /g mL^−1^	Kinematic viscosity /mm^2^ s^−1^	Vapor pressure /Pa	Refractive index (λ = 532 nm)
Water [47], [48]	0.998	1.00	2.34 × 10^3^	1.334
Ethanol [49], [50], [51]	0.789	1.44	5.89 × 10^3^	1.361
PEG 300 [52]	1.124	~85	<1	1.465

The ablated surface of the sample was parallel to the liquid surface (see Fig. S1). The distance between breakdown position and the sharp edge, *l*, was varied by changing the position of the laser spot on the sample surface, as schematically shown in Fig. S1. Chemical composition of the samples was analyzed after the experiments using an X-ray fluorescence spectrometer (Thermo Scientific Niton XL3t GOLDD+) and is shown in Table 2 along with the corresponding sample thickness.

**Table 2:** Thickness and chemical composition of the SS samples used in the experiments.

Sample	Thickness *d* /mm	Chemical composition /wt.%							
		Cr	Ni	Mn	Cu	Mo	V	Si	Fe
S1	0.025	16.0	10.5	1.5	0.35	1.5	0.09	0.50	balance
S2	0.38	18.5	8.1	1.7	0.51	0.38	0.13	0.30	balance
S3	1.0	17.3	8.8	0	0.21	0.1	0.1	2.98	balance
S4	1.6	16.8	10.0	1.8	0.41	2.1	0.11	0.52	balance
S5	2.0	15.3	0.28	0.69	0	0	0	0	balance

### 2.2 Setups for measuring bubble dynamics

Two different experimental setups, one with diffuse flashlight illumination and another with collimated ps-laser-light illumination were used to observe the shock wave and bubble dynamics as well as the dynamics of re-entrant liquid injection that may appear when the bubble expands over the edge of the sample.

**Fig. 1:**
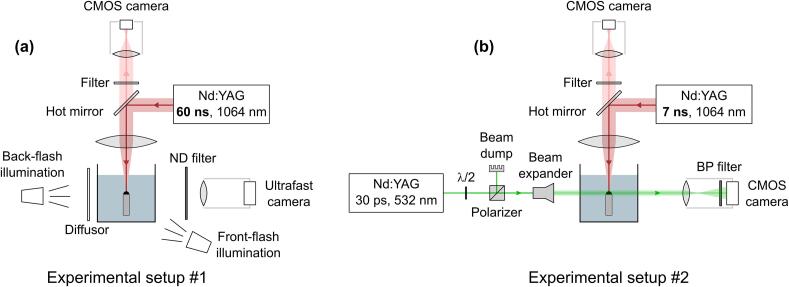
Experimental setups for (a) cinematography by diffuse illumination and for (b) ultrashort shadowgraphy by collimated illumination.

*Setup for cinematography by diffuse illumination* is shown in Fig. 1a, and is labeled as experimental setup #1. It was built and located at the Department of Energy and Hydrocarbon Chemistry, Kyoto University (Japan). As an excitation source we used a Nd:YAG laser with 1064  nm wavelength and pulse duration (FWHM) of 60  ns. The pulses with energy between 10  mJ and 55  mJ were reflected off a hot mirror to enable simultaneous observation of the sample from the top by a CMOS camera (Ximea, MQ022MG-CM, 2048 × 1088 pixel) for positioning. Two photographic flashes with ~1  ms light pulse (the temporal intensity profile is shown in Fig. S2), one from the back and another from the front were used for illumination. A diffusor was placed between the back illumination source and glass cuvette for a more homogenous and diffuse illumination. The bubble dynamics was captured by an ultrafast camera (Shimadzu HPV-2A with Mitutoyo MY10X-803 objective) at 500  kHz frame rate and shutter time of 250  ns. A neutral density (ND) filter was also implemented in front of the objective to reduce overexposure due to bright plasma.

This kind of high-speed cinematography enables the capturing of the whole bubble dynamics within a single shot, but with temporal resolution of only 250 ns, which does not allow to measure some very fast phenomena, such as initial bubble dynamics or a shock wave. As will be theoretically explained in the next section, the diffuse illumination is essential for seeing phenomena *inside* the cavitation bubble.

*Setup for ultrashort shadowgraphy by collimated illumination* is shown in Fig. 1b, and is labeled as experimental setup #2. It was developed and located at the Faculty of Mechanical Engineering, University of Ljubljana (Slovenia) to measure the very early stage (<3  μs) of the bubble expansion, where the bubble wall velocity exceeds 100  m s^−1^. A Nd:YAG laser with 1064  nm wavelength and 7-ns pulses (FWHM) with energy of 10.6  mJ was used as an excitation source. Since the exposure time of the ultrafast camera used for the cinematography within experimental setup #1 is not sufficient to obtain sharp images with resolution below micrometer per pixel, the second harmonic (λ  =  532  nm) of another Nd:YAG laser (Ekspla, Lithuania, PL2250-SHTH) with pulse duration (FWHM) of 30  ps was used for illumination. The images were captured by a CMOS camera (Ximea, MQ013MG-ON, 1280×1024 pixels with Ricoh FL-CC5028-2 M objective) with long exposure (~1  ms), similarly as already described in Ref. [53]. An attenuator, consisting of a half-wave plate (λ/2), polarizing beam-splitter, and a beam dump, was used to set the appropriate intensity of the illumination that was led through a beam expander to enable homogeneous illumination of the observed area. A narrow band-pass (BP) filter (532 ± 10  nm) was placed between the objective and the camera to minimize the irradiation emitted by the laser-induced plasma. Here, the exposure time is defined by the duration of illumination pulse, while the delay between optical breakdown and time of illumination is set by the delay between triggering signals from the signal generator (Tektronix, US, AFG 3102, 1GS/s, 100  MHz) for excitation and illumination lasers. The jitter of this synchronization equals ±0.3  μs, since the excitation laser is passively Q-switched. Thus, the accurate delay between the excitation and illumination pulse was measured by using two photodiodes with 1  GHz bandwidth.

Ultra short exposure time enables the capturing of fast laser-induced phenomena, such as shock waves, but only one image can be acquired from an individual breakdown event. Considering the speed of sound in water (1.5  km s^−1^), the theoretical spatial resolution with 30-ps illumination equals 0.05  μm. However, this approach requires multiple shots to acquire the whole bubble dynamics at different times after the optical breakdown, which calls for high repeatability of the observed phenomena.

## 3 Role of illumination diffusivity in bubble imaging

Optical observation of cavitation bubbles is important for studying acoustic [21], [54], hydrodynamic [55], [56] and laser-induced cavitation [14], [18], [40], [43], [57], [58], as well as to develop different applications including nanoparticle production [8], [9], [28], [30], underwater breakdown spectroscopy [59], enhanced heat transfer with nucleate boiling [60], refrigeration [61], [62], microfluidics [63] and laser biomedical procedures [64]. Understanding the influence of illumination on their perception and interpretation can sometimes prove difficult, since it involves refraction and multiple reflection of light at the interface of media with different optical densities. This section is, therefore, dedicated to a theoretical insight into illumination of a spherical bubble with light sources of different diffusivity. As will be shown, the increased diffusivity is essential for observing liquid injections *inside* the cavitation bubble.

**Fig. 2:**
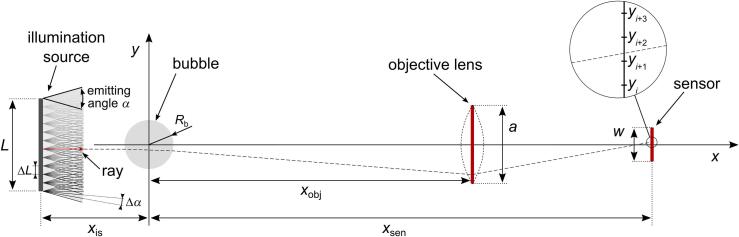
Numerical modeling of illumination in bubble imaging.

**Fig. 3:**
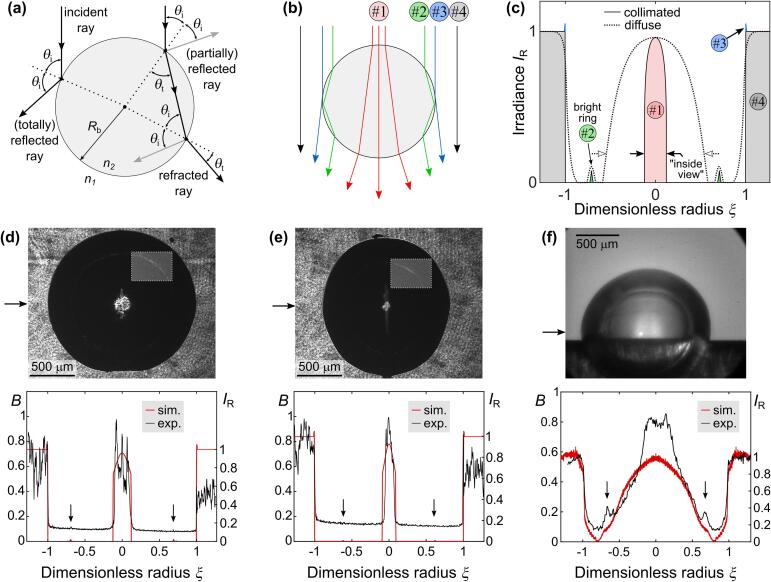
(a) Reflection and refraction of rays at the bubble wall. Angles of incidence and refraction are denoted by *θ*_i_ and *θ*_t_, respectively. (b) Different types of illuminating rays in a vapor bubble with (c) schematically shown corresponding contribution to irradiance profile. (d)–(f) Images of laser-induced cavitation bubbles acquired by experimental setup #2 in (d) water (*n* = 1.33) and (e) PEG (*n* = 1.465) and by (f) experimental setup #1 in water. The arrows on the left-hand side of the acquired images mark the position of the (experimental) irradiance profile in the corresponding bottom graphs, which compare simulation results with experiments. Image brightness *B* is relevant to experimental profiles, while irradiance *I*_R_ is relevant to simulated profiles.

The magnification, resolution, depth of field, and the measuring range of the image acquired by the sensor depend on the properties and relationship between the object, objective lens, and the imaging sensor (schematically presented in Fig. 2). However, special care should be taken when observing media that are transparent for illumination spectrum and contain interfaces between different optical densities [65]. One of such examples is observing two phase phenomena, where the interface between the solid-liquid and/or liquid-gas environment alters the direction of light rays due to light reflection and refraction at the interface, as depicted in Fig. 3a. The perception of such phenomena is, therefore, not as straightforward and requires proper interpretation [66].

Illumination of a bubble for capturing its image by a digital sensor can be modeled as sketched in Fig. 2. Here, the illumination source is located at a distance *x*_is_ from the bubble center. The source of dimension *L* is modeled by discrete point sources (separated by Δ*L*), each of them radiating rays within an emitting angle *α*. The emitting angle is discretized by intervals of Δ*α*. When *α* equals zero, the light source is considered *collimated*. The rays from the illumination source are gathered by an objective lens, located *x*_obj_ from the bubble center, where the lens dimension *a* represents its aperture. An image is acquired by a digital sensor, positioned in the image plane at *x*_sen_ from the bubble center. Numerical values of these parameters, considered in the model, are listed in Table S1 of the Supporting Information.

Even a seemingly simple example of illuminating a *static* vapor bubble that floats inside liquid environment proves difficult to describe analytically due to high complexity of the problem [65]. We, therefore, developed a numerical ray tracing model (Fig. 2 and Section S2 of the Supporting Information) based on the laws of geometrical optics to demonstrate and clarify the effect of illumination on perception of cavitation bubbles. Assuming cylindrical symmetry with respect to the optical axis, the problem is reduced to a plane. The cross-section of the vapor bubble is defined as a circle, while the starting illuminating rays are represented by coplanar lines with desired direction (within emitting angle *α*). Irradiance profile that reaches the sensor is then approximated for different illumination sources considering (total and partial) reflection and refraction of rays at the liquid-gas interface. Intensity of the rays is determined by Fresnel equations [Eqs. (S8)-(S10)].

The bubble with radius *R*_b_ is assumed to contain vapor with refractive index *n*_2_ = 1, while the liquids considered in the simulations are water and PEG with refractive indices *n*_1_ = 1.33 and *n*_1_ = 1.465, respectively. The assumptions and basic principles of the model are presented in higher detail in Section S2 of the Supporting Information.

Fig. 3b and c schematically show the contribution of different types of incident rays to the irradiance profile in case of a collimated (*α* = 0°, solid line) and diffuse (dotted line) illumination source. Illumination rays can be generalized as:

• *type* *#1* *rays* that travel through the bubble and are refracted twice – when entering and when exiting the bubble;
• *type* *#2* *rays* that are reflected inside the bubble (also more than once, as schematically shown in Fig. 3b);
• *type #3 rays* that are reflected at the outer bubble wall; and
• *type #4 direct rays* that do not intersect the bubble.

At this point, it is helpful to define irradiance on the sensor *I*_R_ as the incident irradiance normalized by the average irradiance of the illumination source. Irradiance *I*_R_ of the image background should, therefore, equal 1. Furthermore, for clearer explanation of the observed image, it is convenient to introduce the dimensionless radius *ξ* from the sensor’s center as the distance from the sensor’s center *y* normalized by the bubble radius in the image plane *R*_bi_

(1) $$\xi =\frac{y}{R_{bi}}$$

where *R*_bi_ can be easily calculated as a product of bubble radius *R*_b_ and optical magnification of the system *M*.

As can be seen from Fig. 3c, each of the four generalized rays contributes to a specific part of the irradiance profile, shown in further detail in Fig. S9. Rays that do not intersect the bubble (type #4) form the bright background around the bubble image (|*ξ*| > 1). Considering merely type #4 rays, the emitting angle *α* of the illumination source (in combination with the objective’s aperture *a*) influences the gradient of irradiance profile at the bubble wall, i.e. around |*ξ*|  =  1. As the emitting angle *α* is increased (i.e., the illumination is turned from collimated towards diffuse; dotted line in Fig. 3c), the bubble wall becomes blurred (e.g., see also image in Fig. 3f) due to rays traveling beside the bubble wall not collinearly with the optical axis.

Rays that reflect at the outer bubble wall (type #3) also contribute to the irradiance profile around |*ξ*|  =  1. In case of a collimated source (*α*  =  0°), these rays result in a slight increase of irradiance at the outer side of the bubble interface (|*ξ*|  >  1), albeit the effect is not very pronounced as the collimated rays are quickly reflected out of the aperture of the objective lens. However, as the emitting angle *α* of the source is increasing, more of these reflected rays reach the sensor, leading to increased irradiance also at |*ξ*| < 1 (further detail in Figs. S11 and S12). In this way, the perceived bubble appears smaller [65], which should certainly be considered when the accurate determination of bubble size is the target.

Rays that reflect inside the bubble (type #2) result in appearance of a bright ring inside the bubble. The position of the ring with respect to bubble wall depends on the ratio of refractive indices outside and inside the bubble *n*_1_/*n*_2_. Increasing this ratio causes the ring to appear closer to the bubble center, while increasing the source emitting angle α makes the ring wider.

Finally, the rays that travel through the bubble without reflection (type #1) manifest as bright area in the center of the bubble. These rays are crucial for observing phenomena that occur *inside* the vapor/cavitation bubbles. The diameter of the central illuminated area or “inside view”, schematically marked by horizontal arrows in Fig. 3c, depends both on the ratio of refractive indices, as well as on the emitting angle of the illumination source *α* (in combination with objective’s aperture *a*). In case of collimated source, such as a laser, this diameter is fairly small compared to the radius of the bubble (pink area in Fig. 3c), making observations of fluid dynamics inside the bubble practically impossible. On the contrary, using a diffuse source such as a flashlamp enables the light to enter the bubble at various angles, which significantly increases the observable area inside the vapor bubble.

Modelling of the bubble illumination was validated by comparison of the theoretical and experimental results, shown in Figs. 3d–f. Experimental setup #2 with collimated ps-laser illumination was used to capture images in Figs. 3d, e, while experimental setup #1 with a diffuse light source (utilized by a photographic flash with significantly wider emitting angle and a diffusor) was used for acquiring the image in Fig. 3f. The experimental profiles (black curves in Figs. 3d–f) show image brightness *B* (left-hand scale on y axes) calculated by normalizing the average brightness value of 5 consecutive lines of pixels (locations marked by horizontal arrows in Figs. 3d–f) with 255, since the images were captured in 8-bit gray scale. This yields brightness *B* ranging from 0 (black) to 1 (white). These profiles are compared to the simulated irradiance *I*_R_, where a value of 1 represents the irradiance of illumination source – background.

To show the effect of liquid’s optical density, water (Fig. 3d) with refractive index 1.33 and PEG (Fig. 3e) with refractive index 1.465 were used in the experiments. Influence of the emitting angle of the light source was determined in water, shown in Fig. 3f (with simulated emitting angle *α*  =  46°). Bubbles in Figs. 3d and e were produced in infinite liquid, while the bubble in Fig. 3f was induced on a thin metal sample.

In case of collimated illumination (Figs. 3d, e), the experiments agree with the theoretical simulation very well. The increased noise of the background compared to flash illumination is due to coherence of the laser beam leading to interference effects forming a speckle pattern. Irradiance of the background is decreasing from left to right, as the illuminating laser beam was not perfectly aligned with the optical axis of the imaging system. The measured irradiance of the background (at *ξ*  ≫  1), therefore, differs from the predicted profile, as the model does not account for this. Bright rings that arise from reflections inside the bubble (due to type #2 rays), indicated by vertical arrows in Figs. 3d–f, are detected and their position with respect to the bubble wall is theoretically predicted. For better visibility of the ring, a (rectangular) part of the bubble in Figs. 3d and e is brightened and increased in contrast. The brightness of the ring is fairly dependent on the reflectivity at the bubble wall, since it arises from the rays that reflect inside the bubble (type #2).

Governed by Fresnel relations [Eqs. (S8)–(S10)], reflectivity depends on angles of incidence and refraction, as well as orientation of illuminating-light *polarization* with respect to the plane of incidence. As shown in Section S2 of the Supporting Information (Fig. S10), linear polarization of the illuminating source leads to symmetrical brightness variation of the ring, while background and central illuminated area of the bubble remain fairly similar. The size of the central illuminated area inside the bubble (area #1 in Fig. 3c) also fits the model well. One can see that higher optical density of the liquid (Fig. 3e) decreases the diameter of this area compared to lower optical density (Fig. 3d), while the appearance of the bright ring moves closer to the bubble center. By using collimated illumination in experimental setup #2, the peak of the bright ring (indicated by vertical arrows in Figs. 3d and e) is detected at |*ξ*| ~ 0.69 in water and at |*ξ*| ~ 0.61 in PEG.

On the contrary, it is much harder to closely predict the theoretical irradiance profile in case of diffuse illumination due to difficult characterization of the illuminating source. Albeit the diffusor being placed between the flash and the bubble, the illumination is not homogenious and equally radiant in all angles, which is the model assumption. Furthermore, reflections from surroundings including glass couvette walls as well as object surfaces outside the couvette are significantly greater compared to collimated laser illumination, but neglected in the simulation for sake of simplicity. Nevertheless, we have found that the bright ring from reflections inside the bubble forms in the same position, but is wider compared to laser illumination.

The most important conclusion that follows from the described theoretical modelling of illumination is the confirmation that the bright (i.e., illuminated) central area of the bubble, that is crucial for observation of the phenomena *inside* the bubble, increases by increasing the illumination source diffusivity that is characterized by the emitting angle. Thus, diffuse illumination enables observions of the dynamics of liquid jets inside the bubble (e.g., that occur when the cavitation bubble expands over a sharp edge) with high spatial and temporal resolution.

## 4 Results and discussion

When a high-intensity laser pulse hits the solid-liquid interface (here, it is assumed that the liquid is dielectric and transparent for the excitation-laser light), part of the light is reflected, while the rest is absorbed in the solid. Reflection and absorption depend on the polarization of light, angle of incidence, and refractive indices of the solid target and the liquid. The absorption occurs within a solid-surface layer of thickness that equals the optical penetration depth, defined as *δ*_p_  =   *λ*/4π*κ*. Here, *κ* and *λ* stand for the extinction coefficient of the solid and the wavelength of light, respectively. In metals, the laser beam is absorbed within the skin layer, since the optical penetration depth for metals (at *λ*  =  1064  nm) typically ranges from ~10  nm to ~20  nm.

**Fig. 4:**
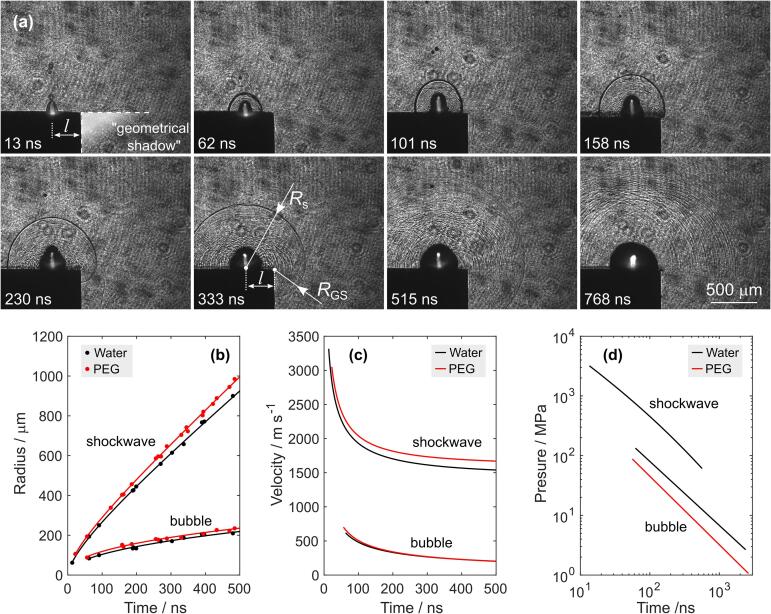
(a) Evolution of the shockwave in water after breakdown of stainless steel at *l*  =  0.3  mm from the edge (experimental setup #2). Geometrical shadow in the first image is intentionally and artificially white-blurred. Temporal profiles of shockwave and bubble wall (b) radius, (c) velocity, and (d) pressure after laser induced breakdown in water and PEG.

The interaction between a nanosecond laser pulse and solid metal results in photon coupling of the electronic and vibrational modes of the target material [67]. The electron–electron coupling leads to increased electron temperature and vaporization of the transiently heated target followed by the expansion of the evaporated atoms, ions, and electrons [68]. The surrounding liquid confines the vapor plume, while the remaining part of the excitation nanosecond-laser pulse further vaporizes the target material, generates additional hot electrons by the absorption of photons, and heats the nascent plasma by inverse Bremsstrahlung [9]. Thus, the plasma plume contains neutral atoms, ions, and electrons from the solid target. The strong confinement by liquid environment results in plasma that is characterized by temperatures of several thousand Kelvins and high pressures up to 10^9^ Pa. The laser-induced plasma adiabatically expands at a supersonic velocity and due to liquid confinement generates a shock wave that propagates into the liquid [69] (see also Figs. 4a and S13), while the recoil during plasma expansion generates an elastic ultrasonic wave within the solid target [70], [71].

As the plasma cools down, it undergoes a phase transition into vapor (cavitation bubble) followed by liquid phase. However, the mechanisms of this transition still remain unclear [9]. At the beginning (<200  ns after the excitation pulse), the cavitation bubble is elongated with the shape similar to the plasma plume outline (see Figs. 4a and S13). When the bubble is induced on a flat target surface, its shape later takes an approximately hemispherical form. If the distances between the bubble center and the edges of this flat surface are significantly larger than the maximum radius of the bubble (*R*_b,max_ ≪ *l*), the bubble expands, collapses, and rebounds several times (typical oscillation time for the bubble induced by ns pulse of several mJ is in the range of several hundred microseconds) and usually ends with long-life (i.e., in the millisecond to second range) persistent microbubbles remaining above the solid surface [9]. In case of irradiating a flat metal surface, the light-to-bubble conversion efficiency is highest when the excitation-beam focus is positioned slightly below the target surface [26]. However, the aim of our experiments is to observe the dynamics when bubble radius is comparable to or larger than the distance between the bubble center and the edge (*R*_b,max_ > *l*), resulting in bubble overflowing the edge of the solid surface (see Fig. S1).

### 4.1 Initial evolution of the shockwave and cavitation bubble

The dynamics of the shock wave and cavitation bubble during the initial several microseconds following optical breakdown in close proximity (distance *l*) to the edge was evaluated using the experimental setup for ultrashort shadowgraphy (experimental setup #2). Since this setup allows acquisition of laser-induced phenomena only in a single time instance, multiple events at different time intervals after the excitation pulse were captured to obtain a temporal evolution. Typical sequence of images with a clearly visible shock wave and cavitation bubble within first eight hundred nanoseconds in water is shown in Fig. 4a, while Fig, S13b shows typical images for PEG (the raw data are available in Ref. [72]).

The measured radii of the observed phenomena as a function of time after irradiation are presented by the dots in Fig. 4b (and Figs. S14 and S16). They were obtained by fitting a circle to the acquired images with 1.6  μm/px resolution. Curves (solid lines in Fig. 4b) defined by Eqs. (S12) and (S15) were fit to discrete experimental measurements of radii in order to obtain continuously derivable functions. The corresponding velocity profiles could then be calculated by simple derivation of these functions (see details in Section S3 of the Supporting Information).

As visible from Figs. 4c and S15, shockwaves in both liquids propagate supersonically with velocity exceeding 2  km s^−1^ at 50 ns after optical breakdown. By time, shockwave velocity converges to the speed of sound, which can be estimated in both liquids as a fitting parameter in Eq. (S12) (see also Table S2). We estimated the shockwave velocities to 1.4  km s^−1^ for water and 1.6  km s^−1^ for PEG. These results are in good agreement with the values reported in the literature, i.e.,1483 m s^−1^ for water [73] and 1615  m  s^−1^ for PEG [74].

Velocity of the shockwave propagation was also investigated in the “geometrical shadow” (see the intentionally shaded area in Fig. 4a at 13  ns). Here, the measuring range is <0.5  mm (i.e., the distance from the sample edge to the bottom of the captured images), while the shockwave velocity is in the order of 1.5  km s^−1^. Thus, the shockwave front in the “geometrical shadow” is observable only within a very narrow time gap of ~0.3  μs, which is difficult to obtain with the jitter of experimental setup #2. Therefore, from the acquired images we can only roughly estimate that the velocity remains similar to the remaining (i.e., outside the geometrical shadow) part of the shockwave. However, the curvature radius of the part of the shockwave front that propagates in the geometrical shadow, *R*_GS_ (see definition in Fig. 4a), is smaller than the radius of the (remaining) shockwave *R*_s_ due to breakdown being induced *away* from the edge. The absolute difference in these radii is constant with time and equals approximately the distance between the breakdown spot and sample edge, i.e., *R*_s_ – *R*_GS_ ~ *l* (see Fig. 4a at 333  ns).

Bubble velocity is difficult to determine during the first 50  ns after the breakdown, as the interface between liquid and vapor is not jet clearly defined due to supercritical state of the liquid. After expansion, when the temperature and pressure decay lead to transition from supercritical to gaseous (vapor) state, bubble wall forms. The first measurable velocity (at 65  ns after optical breakdown) was estimated to 600  m s^−1^ for water and 650  m s^−1^ for PEG (Figs. 4c and S17). It should be noted, that more precise velocity measurements could be collected by using two or more consecutive laser pulses for multi exposure of the shockwave/bubble within several nanoseconds (e.g., by using similar approach as described in Ref. [35]).

As has been shown by Vogel et al., there is a strong correlation between the shockwave velocity *u*_s_ and the shockwave pressure *p*_s_, which can be described by the following relation [14].

(2) $$p_{s}=c_{1}\rho_{0}u_{s}\left(10^{\left(u_{s}-c_{0}\right)/c_{2}}-1\right)+p_{0}$$

In Eq. (2), *c*_0_ stands for the local speed of sound and was determined from the fit [Eq. (S12)], while *p*_0_ is the ambient pressure of the liquid (assumed to be 100  kPa). For water, constants *c*_1_ and *c*_2_ equal 5190  m s^−1^ and 25 306  m s^−1^ [14], respectively. The constants *c*_1_ and *c*_2_ were obtained by Rice and Walsh [75] from the Rankine-Hugoniot relations and an analytical fit of the experimental Hugoniot curve data for water. Due to lack of these constants for PEG, shockwave pressure was only estimated for water and is shown by the black curve in Fig. 4d. As the velocity approaches the speed of sound, the shockwave converts into an acoustic wave and its pressure decreases towards the ambient pressure of the liquid.

The bubble dynamics for incompressible and nonviscous liquid can be described by the Rayleigh-Plesset equation that is derived from the continuity equation and the Navier-Stokes equation as [76]

(3) $$\Delta p=\rho_{0}\left(\frac{3}{2}{\overset{˙}{R}}_{\text{b}}^{2}+R_{b}{\overset{¨}{R}}_{b}\right)$$

In Eq. (3), *R*_b_ stands for the bubble radius as a function of time, dot represents a time derivative, and *ρ*_0_ is the density of the liquid. The pressure difference at the bubble wall Δ*p* equals

(4) $$\Delta p=p_{b}-p_{0}$$

where *p*_0_ denotes the pressure of the surrounding liquid and *p*_b_ is the pressure at the bubble wall that can be expressed by the bubble vapor pressure *p*_v_ as $p_{\text{b}}=p_{\text{v}}-2\sigma /R_{\text{b}}-4\eta {\overset{˙}{R}}_{\text{b}}/R_{\text{b}}$. The terms corresponding to the surface tension *σ* and viscosity *η* can be neglected, as they do not play a significant role in bubble dynamics in case of millimeter-scale bubbles [77]. Thus, the pressure difference at the bubble wall in Eq. (3) simply equals Δ*p* = *p*_v_ – *p*_0_.

We calculated the bubble pressure difference by deriving the temporal bubble radius evolution (obtained by fitting Eq. (S15) to discrete radius measurements) and incorporating the first and second derivative into Eq. (3), as described in Section S3.2 of the Supporting Information. By doing this, the initial pressure difference at the cavitation bubble wall (at 65  ns after breakdown) could be estimated to 1.3 × 10^3^ bar in water and 0.7 × 10^3^  bar in PEG (see Figs. 3d and S18). These results are found consistent with the work of Vogel et. al, who studied optical breakdown in infinite water [14]. Similarly, Lam et al. [77] and De Giacomo et al. [28] calculated the vapor pressure inside the bubble on the basis of temporal radius evolution that was measured by ultrafast videography. Due to longer time intervals between consecutive images, their first measurements begin at 5  μs after excitation, at which time the estimated pressure *p*_v_ is in the order of 100  bars, presuming constant pressure of surrounding liquid *p*_0_. In comparison, our results do not make this assumption but rather consider the overall pressure difference Δ*p* = *p*_v_ – *p*_0_ at the bubble wall. However, since the surrounding pressure *p*_0_ is usually assumed in the order of several bars [77], the difference between *p*_v_ and Δ*p* (assuming constant *p*_0_) should only be noticeable when pressure inside the bubble decreases below 100  bars. Taking this into account, our results propose the decrease of pressure difference to the value of 100  bars sooner (compared to [28], [77]), i.e., 700  ns after excitation in water and 400  ns in PEG. The inconsistency could perhaps be attributed to significantly higher temporal resolution and number of images obtained in the first microsecond with our setup, which increases the validity of radii measurements during that short initial period.

### 4.2 Development of the cavitation bubble on finite geometry

The majority of existing LAL studies on solids deal with “infinite” flat geometries of the ablated surfaces [20], [32], [77], [78], where the maximum bubble radius is significantly smaller than the distance between the bubble center and the nearest edge of the flat solid surface (*R*_b,max_ ≪ *l*). On the contrary, in this study we deliberately induce breakdown in close proximity to the edge of the flat surface in order to study phenomena on finite geometry. We achieved this by either *(i)* irradiating a very thin sample (Fig. S1a) or *(ii)* positioning the breakdown spot close to the sample’s edge (Fig. S1b). In the first case, 17 mm wide samples with thicknesses from 25  μm to 2  mm were clamped in an “H” configuration (see Fig. S1a). Thus, the direction of the longer (17 mm) dimension remained “infinite” (as it was significantly larger than the maximum radius of the bubble), while the shorter dimension could be classified as “finite”.

In the second case, samples were clamped in an “L” configuration (Fig. S1b). Sample thickness was kept constant and equaled *d*  =  2  mm, while the breakdown was positioned between *l*  =  0.1  mm and *l*  =  1.7  mm away from the edge of the sample (dimension *b* in Fig. S1b). The bubble thereby reached three (out of four) edges of the sample. Samples with thicknesses over 2  mm were deliberately not used to avoid the shadow resulting from misalignment of the sample surface with the optical axis.

**Fig. 5:**
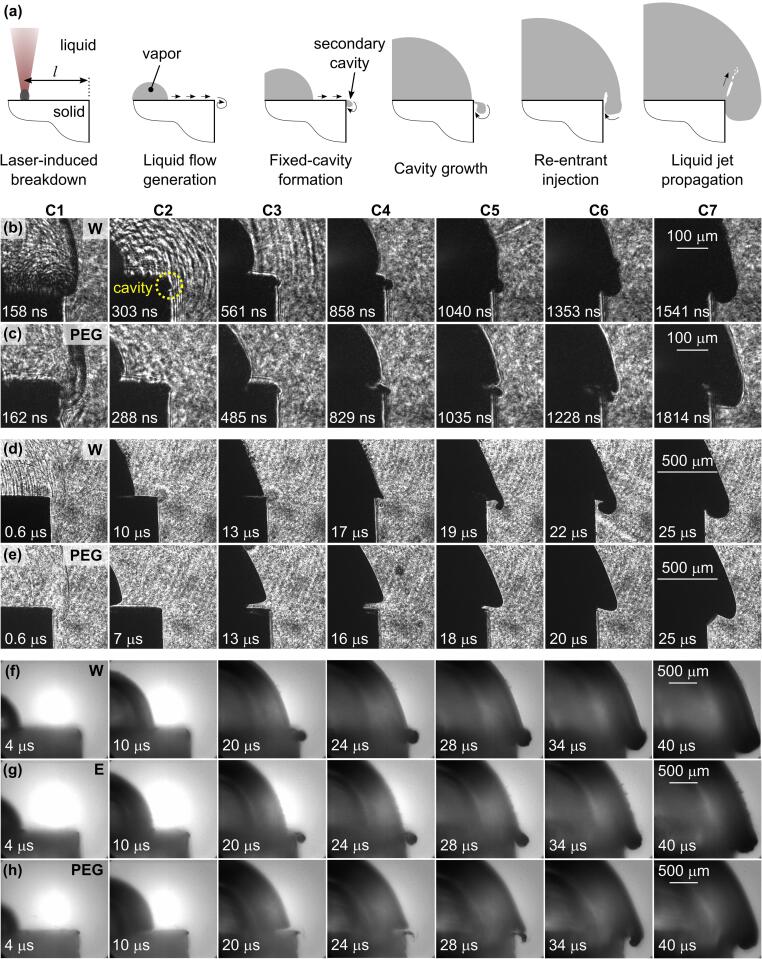
(a) Schematic presentation of the bubble-induced flow evolution over a “cliff-like” 90° edge in case of secondary cavity formation. Bubble overflow was induced at (b), (c) *l*  =  0.3  mm, (d), (e) *l*  =  1  mm, and (f)–(h) *l* = 1.7  mm from the edge in water (W), ethanol (E), and PEG. Pulse energy equals 10.6  mJ for (b–e) and 55  mJ for (f–h).

Generally, when the bubble is produced far away from the edge of the solid sample, its radius at maximum size, *R*_b,max_, is smaller than the distance from the bubble center to the edge (*R*_b,max_ ≪ *l*). In that case, the bubble does not reach the edge and the sample surface can be considered as infinite. Contrarily, when the edge is located in close proximity to the breakdown spot (*l* < *R*_b,max_), the liquid–vapor interface (i.e., the bubble wall) expands beyond the solid edge and results in overflow of (first) the liquid that surrounds the bubble and (later) the vapor that is inside the bubble. During the liquid flow (driven by the expansion of the cavitation bubble) over the edge, a secondary bubble (i.e., the so-called secondary cavity) can form just beyond the edge due to formation of a low pressure area in the liquid. Furthermore, a jet of liquid can in some cases penetrate into the laser-induced cavitation bubble when the bubble overflows the edge (see schematics in Fig. 5a). Development of such injection depends on *(i)* the energy of the bubble (which determines its maximum radius) and *(ii)* the distance between the bubble center and the edge, as well as *(iii)* the physical properties of the liquid.

Since the maximum bubble radius, *R*_b,max_, and the distance of the bubble from the edge, *l*, are correlated, it is appropriate to introduce the dimensionless distance from the edge as

(5) $$\zeta =\frac{l}{R_{\text{b,max}}}$$

However, in our experiments, we were not able to determine the maximum radii of the bubbles with existing experimental setup due to high optical magnification that resulted in smaller viewing area than the bubble at its maximum size. Thus, when needed, *R*_b,max_ was roughly estimated from the collapse time of the bubble *T*_c_ as [40], [79]

(6) $$R_{\text{b,max}}=\frac{T_{\text{c}}}{0.915\sqrt{\rho_{0}/\left(p_{0}-p_{\text{v}}\right)}}$$

In Eq. (6), *p*_v_ stands for the vapor pressure inside the bubble.

In order to investigate the effects and dynamics of liquid–vapor overflow over a finite geometry, we generated the bubble at different distances *l* from the edge. In this case, samples S4 and S5 (see Table 2) were used for the experiments with experimental setups #2 and #1, respectively. During all the measurements, we have not detected any movement of the bubble center (the raw data are available in Ref. [72]).

**Fig. 6:**
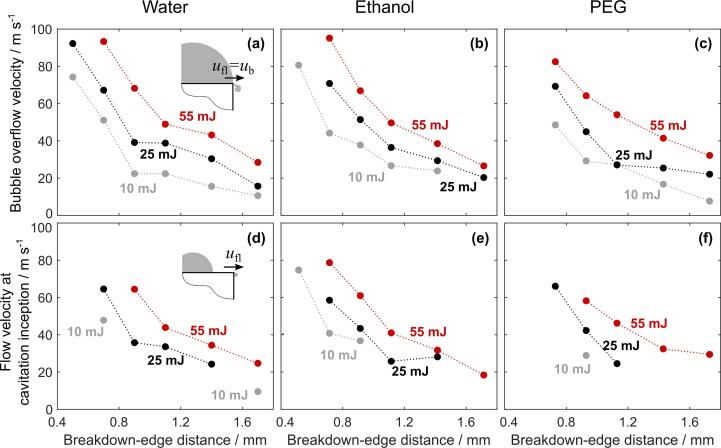
The bubble overflow velocity over the sharp edge in (a) water, (b) ethanol, and (c) PEG at different breakdown-edge distances and pulse energies (marked in each subfigure). (d-f) Velocity of the liquid flow over the edge at the time of the secondary cavity inception in water, ethanol, and PEG, respectively.

Typical images of bubble expansion induced at different distances from the edge of the flat surface and in different liquids (water, ethanol, and PEG) are shown in Fig. 5. After the laser-induced breakdown, the expanding bubble generates a liquid flow that is “guided” by the surface of the sample (first two sketches in Fig. 5a). When the liquid overflows the edge of the solid, a low pressure area forms beyond the edge. If the pressure in this area decreases below the vapor pressure of the liquid, vaporization of the liquid occurs. This results in development of a (vapor filled) secondary cavity (third sketch in Fig. 5a) by breaking away of the flow from the guiding surface at the point of low-pressure. Flow velocity in this instant is estimated in Fig. 6d–f. From this point, the main flow of the liquid follows a free trajectory that is determined by the pressure field and usually returns to the surface at some point downstream. The space between the solid-guiding surface and the free-liquid surface is generally classified as a fixed cavity [80], [81], [82], since its position – with respect to the rigid boundary upon which it forms – is fixed.

The flow of liquid in most experimental studies of fixed cavitation [82], [83] is continuous and, therefore, a somewhat cyclical process develops. The fixed cavity typically undergoes three phases [80], [81]: *(i)* formation and growth, *(ii)* filling, and *(iii)* breakoff. It is generally accepted [84] that cavity breakoff is caused by re-entrant injection of liquid, which forms in the high-pressure area at the downstream end of the cavity and flows upstream towards the leading edge. The upstream velocity of this re-entrant jet is proportional to the velocity of the liquid at the cavity interface.

**Fig. 7:**
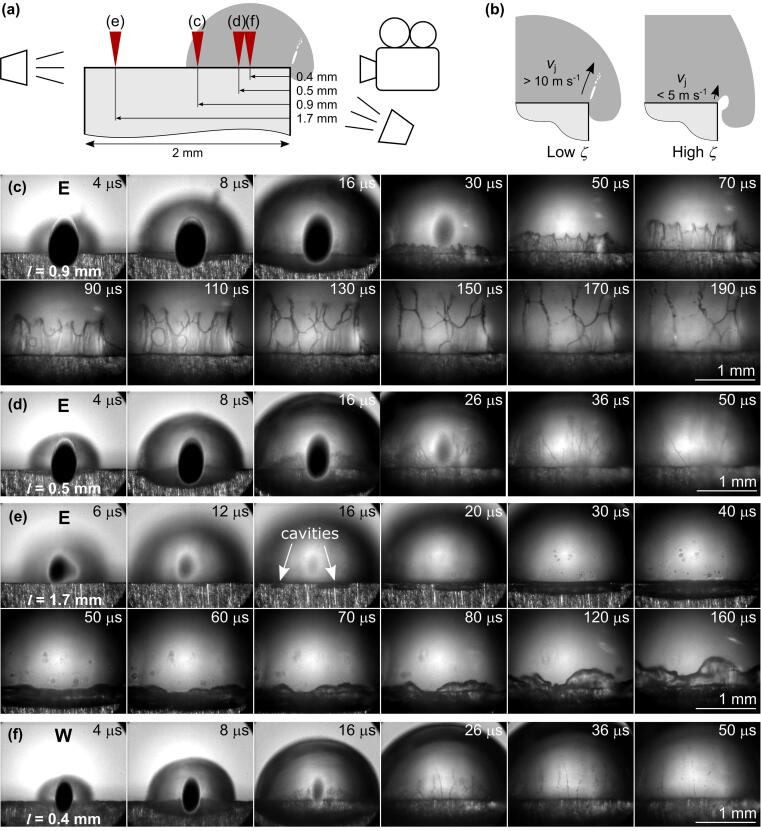
(a) Schematics showing the breakdown position with respect to the edge. (b) Schematic representation of injection dynamics in case of lower *ζ* (breakdown closer to the edge) and higher *ζ* (breakdown farther from the edge). (c–e) Injection of ethanol (E) and (f) water (W) inside the bubble following bubble’s expansion over the edge. The breakdown-edge distance *l* is shown in (a). Pulse energy equals 25 mJ. Black spots in the center of the bubble are due to saturation of the ICCD shortly after plasma formation.

When the flow of supplying liquid is continuous, cloud cavitation [85] is often observed downstream of the attached cavitation, resulting from the abovementioned periodical cavitation process. Contrarily, the flow driven by expansion of the bubble is not continuous, since the supply of liquid is discontinued when the bubble expands over the edge. Consequently, instead of recombining with the main flow and causing cavity breakoff, re-entrant jet propagates further through the vapor bubble, as schematically presented by the fifth and sixth sketch in Fig. 5a (this injection can be clearly observed by using diffuse illumination – Fig. 7). The secondary cavity in the meantime merges with the cavitation bubble, which continues to expand away from the edge.

Experimental observations in water and PEG reveal that the secondary cavity is formed ~300  ns after the optical breakdown that is induced by 10.6-mJ laser pulse at *l*  =  0.3  mm (Figs. 5b and c, column C2). Due to significantly higher viscosity of PEG compared to water, bubble detachment from the guiding surface (Fig. 5c, columns C4 and C5) becomes more apparent in PEG than in water, which is consistent with findings of Hupfeld et al. [86]. Bubble wall reaches the edge in both liquids around 0.8–0.9  μs after the excitation (see Fig. S16). According to temporal velocity profiles in Fig. 4c (see also Fig. S17 for larger timescale), the velocity of the bubble wall at that time equals around 150  m s^−1^.

Fig. 5d, e show the bubble dynamics for the same laser pulse energy, but at increased breakdown-edge distance, *l* = 1 mm. Increasing the distance results in lower velocity of the liquid flow (induced by the same pulse energy) over the edge of the sample. Based on the images in Figs. 5d, e, velocity of the liquid when the bubble overflows the edge is estimated to ~30  m s^−1^. Due to lower flow velocity (compared to *l*  =  0.3  mm), the pressure behind the edge is no longer sufficiently low to vaporize the liquid. Consequently, the secondary cavity does not form in this case. However, the dynamics of the bubble wall is still affected. In case of water (having low viscosity), the liquid–vapor interface close to the edge follows a spiral like trajectory (Fig. 5d, column C5), while significantly higher viscosity of PEG leads to more gradual “pulling” of the bubble into the low pressure area (C5 in Fig. 5e). The gap resulting from detachment of the bubble wall from the sample surface (C4 in Fig. 5e) is even more apparent at larger breakdown-edge offsets, since the sheer flow between the sample surface and the bubble wall is maintained for a longer duration of time. The gap is also briefly noticeable in (less viscous) water (C2 and C3 in Fig. 5d) just before it is overshadowed by the bubble’s expansion beyond the edge.

By increasing the energy of excitation pulse from 10.6  mJ to 55  mJ, the secondary cavity forms even if the breakdown-edge distance is increased to *l*  =  1.7  mm (see Figs. 5f–h, as well as Videos S1 and S2 that correspond to images in Figs. 5f and 5h, respectively). This dynamics was captured from a single event (by using experimental setup #1). There is no apparent difference between water and ethanol (Figs. 5f, g), since their properties – especially the vapor pressure and viscosity – are in the same order of magnitude (see Table 1). On the other hand, the secondary cavity that forms in PEG (C4 in Fig. 5h and Video S2) appears thinner and elongated in the direction of the flow. This could be attributed to significantly (three orders of magnitude) lower vapor pressure and significantly (for almost two orders of magnitude) higher viscosity of PEG compared to water and ethanol.

Video S1Secondary cavity formation in water.
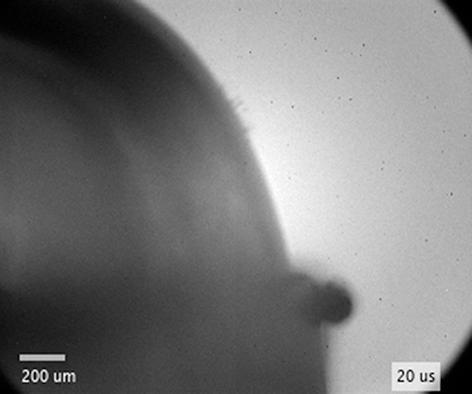

Video S2Secondary cavity formation in PEG.
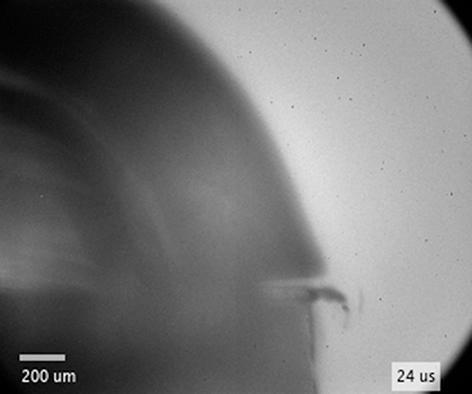

Observation of bubble dynamics from a single event by using experimental setup #1 allows direct estimation of the velocity of liquid flow over the edge *u*_fl_ (Fig. 6). Here, the bubble wall was tracked to determine its velocity *u*_b_. Considering conservation of mass and assuming incompressible liquid, liquid flow at the edge *u*_fl_ can be determined as

(7) $$u_{\text{fl}}=u_{\text{b}}\frac{R_{\text{b}}^{2}}{l^{2}}$$

Fig. 6a-c show the velocity of the *bubble* when it overflows the edge (see schematics in Fig. 6a) in water, ethanol, and PEG, respectively. Since the bubble radius *R*_b_ at this instant equals *l*, bubble wall velocity *u*_b_ (at this moment) is also equal to the velocity of liquid flow over the edge *u*_fl_. These results show that increasing the pulse energy increases the velocity of the liquid overflow in the moment that the bubble overflows the edge. Furthermore, it is also clear that this velocity is decreasing by positioning the breakdown farther from the edge (increasing *ζ*). As *ζ* → 1, the overflow velocity approaches 0, while for *ζ* → 0, the flow velocity at the edge should converge to the very early-stage bubble wall velocity, i.e., several hundred meters per second (see Fig. 4c). However, this cannot be measured by experimental setup #1, since the measurement of bubble wall velocity requires two consecutive frames with a visible bubble wall. Thus, flow velocity could not be determined for the very short breakdown-edge distances, where the bubble wall was visible for less than two frames before overflowing the edge.

Fig. 6d–f present the *liquid* overflow velocity at the moment that the *secondary cavity* (cavitation inception) is visually detected in the acquired videos (see schematics in Fig. 6d). Dynamics during a ±4  μs time window surrounding the secondary cavity formation is shown in Section S4 of the Supporting Information. From our observations, secondary cavitation was only detected if the liquid overflow velocity exceeded ~20  m s^−1^. An exception is detected in case of 10  mJ pulse excitation at *l*  =  1.7  mm in water (Fig. 6d). In this case, preexisting small bubbles on the sample surface evidently act as cavitation nuclei, reducing this threshold velocity (see raw data in Ref. [72]). Calculated flow velocity at the point of inception increases at shorter breakdown-edge distances due to a very rapid rise of the bubble wall velocity during bubble formation. Since cavity formation (vaporization) is not instantaneous, the flow velocity at the instance of secondary cavity detection (only a few microseconds after the laser-induced breakdown) is already significantly above the threshold value.

Interestingly, experiments performed with experimental setup #2 in water and PEG at 1 mm breakdown-edge distance (Figs. 5d, e) did not result in secondary cavity formation, even though the estimated flow velocity at the time of the bubble overflow equaled ~30  m s^−1^. This could perhaps be explained by a different process of sharp-edge preparation (milling in case of samples for experimental system #1 and cutting & polishing in case of experimental system #2), yielding a slight variation of the burr. Results by Petkovšek et al. [55] show that incipient cavitation significantly depends on surface microstructures. Thus, changes in the burr could influence the secondary cavity inception by altering the local flow dynamics and consequently affecting the pressure difference induced at the edge.

### 4.3 Liquid injection into the cavitation bubble

**Fig. 8:**
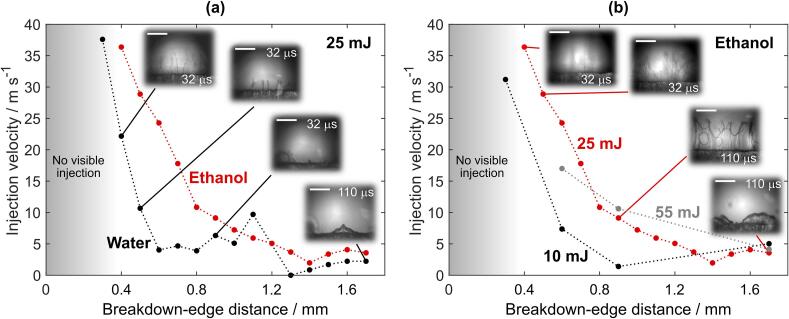
Velocity of liquid injection inside the bubble, induced at different breakdown-edge distances. (a) Comparison of injection velocity in water and ethanol at constant pulse energy of 25 mJ. (b) Comparison of different pulse energies in ethanol. Note that the red curves in (a) and (b) are the same curve. Inset images in (a) and (b) correspond to injections in water and ethanol (at 0.4 mm, 0.5 mm, 0.9 mm, and 1.7 mm breakdown-edge distances), respectively. Scale bars equal 0.5 mm and time indicates the time after breakdown. (For interpretation of the references to colour in this figure legend, the reader is referred to the web version of this article.)

By incorporating diffuse illumination to see inside the cavitation bubble, we were able to detect and analyze the propagating liquid jet at various conditions. Qualitative characteristics of liquid jets are summarized in Fig. 7, while their velocities are shown in Fig. 8. These experiments were performed by experimental setup #1 in water, ethanol, and PEG. However, injections of liquid into the bubble were only detected in water and ethanol, presumably due to significantly higher viscosity of PEG (see Fig. S30 for direct comparison), which substantially decreases the velocity of re-entrant injection. At pulse energy of 25  mJ, liquid injections are observed in water when the breakdown-edge distance exceeds the threshold distance *l* ≥ 0.3  mm, while in ethanol the jets are not visible until this distance increases to *l* ≥ 0.4  mm.

The most pronounced injections were detected in *ethanol* with pulse energy of 25  mJ at *l*  =  0.9  mm (Fig. 7c and Video S3). In this case, velocity and direction of injection shortly after detachment from the edge are fairly uniform from left to right, creating the appearance of a liquid “wall” (Fig. 7c, 70  μs). As the liquid continues to propagate through the cavitation bubble, surface tension transforms this wall into narrow jets and droplets (Fig. 7c, 170  μs). Observation from the side (supplementary Fig. S29 at 120  μs and Video S4) reveals that the direction of injection inside the bubble is in fact not coplanar with the vertical surface of the sample. It is rather directed at a slight angle that points away from the breakdown position, as depicted in the sixth sketch in Fig. 5a. The “tip” velocity of the injection *v*_j_ is estimated to ~10  m s^−1^ (Fig. 8a).

Video S3Front view of injection in ethanol.
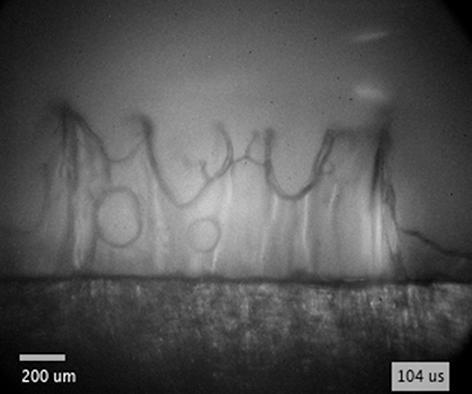

Video S4Side view of injection in ethanol.
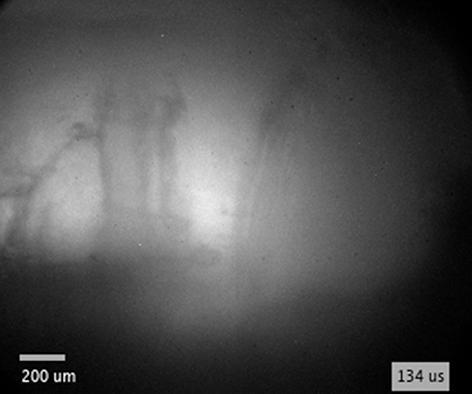

Video S5Injection at lower *ζ* (narrow jets and droplets).
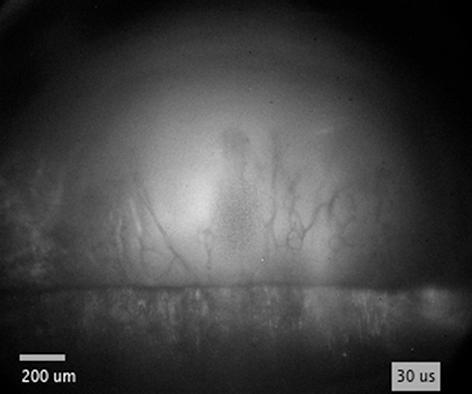

By changing the breakdown-edge distance, characteristics of injection change. Moving the optical breakdown *closer to the edge* (Fig. 7d and Video S5) increases the velocity of the jet, while its shape no longer resembles a wall. Injection velocity in Fig. 7d is estimated to *v*_j_ ~ 30  m s^−1^ (Fig. 8a). The jet in this case rather consists of very narrow jets and droplets that, compared to Fig. 7c, now “originate” in the “central” part of the bubble and propagate also in the horizontal directions (Fig. 7d, 36  μs). The latter can be explained by smaller radius of the bubble at the moment it reaches the edge (i.e., when *R*_b_ ~ *l*), which promotes gradual opposed to simultaneous overflow of the bubble wall along the edge of the sample.

Maximum velocity of injection in water of *v*_j_ ~ 38  m s^−1^ was detected at *l*  =  0.3  mm (Fig. 8a). In ethanol, highest velocity of the jet equal to *v*_j_ ~ 36  m s^−1^ and was observed at *l*  =  0.4  mm. When breakdown-edge distance was decreased below these values (*l* < 0.3  mm in water, Fig. S31a; *l*  <  0.4  mm in ethanol, Fig. S31b, both at pulse energy 25  mJ), the injection was not visible anymore. This might be due to significantly lower volume of injection and its dispersion into smaller droplets with high velocity (possibly exceeding 40  m s^−1^) that makes such detection more difficult – as the image contrast is limited due to necessity of diffuse illumination to see inside the bubble.

Another possible explanation is that the bubble wall reaches the edge too early – when the pressure inside the bubble is still too high to allow the formation of re-entrant jet. Furthermore, smaller radius and, therefore, height of such early stage bubble at the time of (edge) overflow could cause the re-entrant injection to hit the bubble wall instead of propagating through the bubble during its expansion.

Injections were also studied with bubbles being induced in the middle of a thin (25  μm–2  mm wide, see Table 2) sample surface to produce symmetrical “enwrapping” of the solid. Similarly, the injections in such experiments were not visible at thicknesses of the sample below a certain value (<1  mm in water at 25  mJ), leading to similar conclusions (see Section S6 of the Supporting Information).

As the breakdown is induced *farther from the edge*, *ζ* increases and the velocity of the injection *v*_j_ decreases, as visible in Fig. 8a. As can be seen from Fig. 7e (see also Video S6), the jet is practically floating inside the bubble (*v*_j_ ~ 4  m s^−1^) without advancing toward the bubble wall. This can be explained by lower velocity of the bubble wall (and consequently liquid flow) at the time of edge overflow (Fig. 6b), which also reduces the velocity of re-entrant injection. Comparison to jet dynamics induced at lower *ζ* (Figs. 7c, d) is schematically depicted in Fig. 7b. Increasing the breakdown-edge distance also prolongs the time interval before the bubble wall reaches the edge of the sample, which leads to longer exposure of surrounding liquid to low pressure. The secondary cavity thereby grows larger in diameter and length along the edge (Fig. 7e, 16–40  μs) compared to shorter breakdown-edge offsets. The nuclei that initiate the growth of the secondary cavity at inception were generally found to occur at multiple positions along the edge of the sample (Fig. 7e, 16  μs). Whether they primarily originate from the liquid flow or specific spots on the sample surface/edge was not investigated. However, we detected that small bubbles on the surface remaining from previous experiments in some cases act as nuclei (see raw data in Ref. [72], video of 10  mJ excitation in water at *l*  =  1.7  mm) and promote formation of a secondary cavity also at higher ζ. The dark spots that are visible at the bubble wall in Fig. 7e at 40  μs are ripples, most likely caused by droplets that originate from the liquid injection at the farther edge of the sample.

Video S6Injection at higher *ζ* (floating injection).
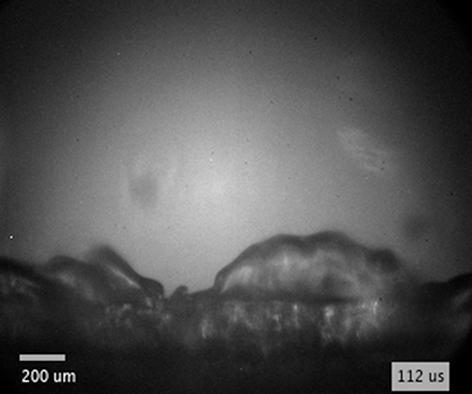

Fig. 8 shows comparisons of injection velocities, *v*_j_, that were observed at different parameters. The data were obtained by manually tracking the “tip” of each injection at three different locations along the edge (raw data in Ref. [72]). The velocity values presented in the graphs are the average of these three measurements.

As can be seen from Fig. 8a, liquid jets in *water* (Fig. 7f) at the same pulse energy were detected at shorter breakdown-edge distances (already at *l*   =  0.3  mm) compared to ethanol. Furthermore, the measured velocity of the jet *v*_j_ at equal breakdown-edge distances is generally lower in water than ethanol (Fig. 8a). The outlying exception at *l*  =  1.1  mm can be explained by slightly asymmetrical bubble overflow of the edge, which resulted in formation of a small injection with relatively high velocity at one side of the edge (see Fig. S32). The inset images of injections induced at equal breakdown-edge distances in water (insets in Fig. 8a) and ethanol (insets in Fig. 8b) additionally reveal that the shape of the injection in water never resembled a “wall” (for full dynamics see Fig. S33). This is most probably due to higher surface tension. Further investigation of underlying causes for differences in jet dynamics induced in water and ethanol is out of the scope of this work. However, in addition to the properties of the liquid, the energy conversion efficiency from the optical pulse energy into the cavitation bubble energy is also important, since it directly influences the maximum bubble radius [26], [40] and, consequently, the ratio *ζ*.

Changing the *laser pulse energy* (Fig. 8b) exhibited a similar effect as variation of breakdown-edge distance. Increasing solely the pulse energy resulted in higher injection velocity, while the jets became narrower (see Figs. S34 and S35 for comparison). Thus, increasing the pulse energy leads to similar results as decreasing the breakdown-edge distance at the same pulse energy. However, at higher pulse energy, probability of a double breakdown due to impurities in the liquid increases. When this happens, the cavitation bubble energy is decreased. As a result of double breakdown (see Fig. S36 for dynamics), jet velocity in case of 55  mJ excitation in ethanol at *l*  *=*  0.6  mm is lower compared to 25  mJ excitation at the same breakdown-edge distance (Fig. 8b).

The similarity of pulse-energy and breakdown-distance effects further demonstrates the importance of dimensionless parameter *ζ*, defined by Eq. (5). By using Eq. (6), we estimated the maximum bubble radii in ethanol (as the most noticeable injections were detected there) to ~1.5  mm, ~1.8  mm, and ~2.3  mm at 10  mJ, 25  mJ, and 55  mJ pulse energy, respectively. From these we can conclude that the injections in ethanol were not visible at *ζ* < 0.2, while the most pronounced jets (Fig. 7c) were detected at *ζ* ~ 0.5.

## 5 Conclusions

We have theoretically and experimentally demonstrated that diffuse illumination (compared to collimated illumination) represents an excellent approach for increasing the observability of fluid dynamics *inside* the cavitation bubbles in the visible spectrum. Thus, diffusive illumination was further used to study the fluid dynamics inside the laser-induced cavitation bubble, when it expands over a 90° sharp solid–liquid boundary, while a high-speed shadowgraphy with ps illumination was used to understand the bubble dynamics within the first several hundred nanoseconds after the laser-induced breakdown. The presented results lead to the following conclusions:

• Bubble-driven overflow of the surrounding liquid in case of a “cliff-like” 90° solid edge may lead to formation of a fixed-type secondary cavity behind the edge. Here, higher pulse/bubble energy and/or breakdown that is induced closer to the edge increase the pressure drop behind the edge and make the conditions more favorable for secondary cavity formation. Secondary cavitation was detected when the liquid overflow velocity exceeded a threshold value of ~20  m s^−1^. Larger cavities were observed at larger breakdown-edge offsets due to longer-lasting cavity growth (vaporization). In case of insufficient bubble energy and/or excessive breakdown-edge distance, the bubble wall is merely “pulled” into the low pressure area without development of a secondary cavity. The trajectory of this overflow depends on the viscosity of the liquid.
• Re-entrant injection of liquid with velocity of up to ~40  m s^−1^ inside the cavitation bubble was clearly observed when the bubble is passing over the 90° edge of the solid sample. The obtained results prove that the jet is far more likely to occur in water and ethanol than polyethylene glycol, which indicates a significant role of liquid viscosity and surface tension. The most pronounced injections were observed in ethanol when the breakdown-edge offset equaled approximately half of the maximum bubble radius. In this case, the shape of the liquid injection firstly resembles a wall. During subsequent evolution (inside the bubble), surface tension of the liquid leads to transition of this liquid wall into narrower jets and droplets.
• Experiments on 25  μm–2  mm thick metal foils reveal the existence of a “threshold” thickness of the sample at which the injections become noticeable. For nanosecond pulses with 25  mJ pulse energy in water, this thickness is in the order of several hundred micrometers when a 90° edge of the sample is considered.

The above-listed conclusions represent new fundamental insights into dynamics of laser-induced cavitation bubbles that expand near a sharp solid–liquid boundary. Such boundary conditions in combination with diffuse illumination that allows observation *inside* the cavitation bubble can be utilized in controlling and improving the yield of nanoparticle production. Furthermore, they serve to a more general understanding of bubble dynamics within confined geometries.

## 6 Funding sources

This research was funded by the Slovenian Research Agency (Projects No. J2-1741 and BI-JP/18-20-004 and research core funding No. P2-0392) and JSPS under the Japan-Slovenia Research Cooperative Program and JSPS KAKENHI Grant Number JP20H02764.

## CRediT authorship contribution statement

**Matej Senegačnik:** Conceptualization, Methodology, Software, Validation, Formal analysis, Investigation, Data curation, Writing - original draft, Writing - review & editing, Visualization. **Kohei Kunimoto:** Investigation. **Satoshi Yamaguchi:** Investigation, Resources. **Koki Kimura:** Investigation. **Tetsuo Sakka:** Conceptualization, Methodology, Investigation, Writing - review & editing, Supervision, Project administration, Funding acquisition. **Peter Gregorčič:** Conceptualization, Methodology, Validation, Formal analysis, Investigation, Data curation, Writing - original draft, Writing - review & editing, Supervision, Project administration, Funding acquisition.

## Declaration of Competing Interest

The authors declare that they have no known competing financial interests or personal relationships that could have appeared to influence the work reported in this paper.
