# Supplementary material for: Dynamics of laser-induced cavitation bubble during expansion over sharp-edge geometry submerged in liquid – an inside view by diffuse illumination
Source: Ultrason Sonochem. 2021 Jan 9;73:105460. doi: 10.1016/j.ultsonch.2021.105460 (PMC8027904; doi:10.1016/j.ultsonch.2021.105460)
Supplement: Supplementary data 1 [file mmc1.pdf]

Supporting Information

Dynamics of laser-induced cavitation bubble during expansion over sharp-edge geometry submerged in liquid – an inside view by diffuse illumination

Matej Senegačnik[1], Kohei Kunimoto[2], Satoshi Yamaguchi[2], Koki Kimura[2], Tetsuo Sakka[2], Peter Gregorčič[1]*

1- Faculty of Mechanical Engineering, University of Ljubljana, Aškerčeva 6, 1000 Ljubljana, Slovenia

2- Department of Energy and Hydrocarbon Chemistry, Kyoto University, Nishikyo, Kyoto 615-8510, Japan

Corresponding author:

** E-mail: peter.gregorcic@fs.uni-lj.si (Peter Gregorčič)*

Table of Contents

S1 Sample positioning and illumination

Fig. S1. *Breakdown positioning in case of bubble overflow observation (a) from the front ("H" type clamping of the sample) or (b) from the side ("L" type clamping of the sample). The camera is located at the opposite side of the sample as illumination. Black lines in the corresponding acquired images represent 500 μm.*

Fig. S2. *Temporal intensity profile of diffuse illumination source (flashlamp) that is used in experimental setup #1.*

S2 Ray tracing model

Fig. S3. *Determining points of intersection with the bubble wall.*

Fig. S4. *(a) Total reflection and (b) refraction of a light ray at the bubble wall.*

Fig. S5. *Reflectivity of light for different angles of incidence when passing from (a) cavitation bubble (water vapor) into liquid (water) and (b) vice versa.*

Fig. S6. *Determining new rays after reaching the intersection point. Reflectivity R is determined from the Fresnel relations considering polarization orientation.*

Fig. S7. *Parameters in ray transfer matrix of objective lens.*

Fig. S8. *Ray tracing simulations of collimated illumination with 50 parallel initial rays. (a) s- and (b) p-polarizations are considered. Brightness of the lines corresponds to relative intensity of the ray, ranging from 0 (white) to 1 (black). Characteristic rays of types #1, #2, and #4 are presented in (a) with red, green, and pink color, respectively.*

Fig. S9. *(a) Sections (intervals) of collimated illumination source that contribute (by superposition) to (b) the total irradiance profile of the vapor bubble in water. Individual contribution of ray types #1-#4 is shown in (c)-(f), respectively.*

Fig. S10. *Images of the same static air bubble acquired by (a) p-polarized and (b) s-polarized collimated (laser) back illumination (experimental setup #2). Brightness and contrast of the image are significantly increased in (c) and (d), respectively, for better visibility of the bright ring. Image brightness profiles, deducted from (a) and (b) are shown in (e) and (f), while simulated irradiance profiles are presented in (g) and (h), respectively.*

Fig. S11. *(a) Ray tracing simulation of collimated illumination incident at 45° (with respect to the horizontal optical axis). Images of the same static air bubble are acquired by collimated back illumination with the angle of incidence equal to (b) 0° and (c) 45° (from the left) with respect to the optical axis. (c) Image brightness profile deducted from (b) and (c) at locations that are marked by the horizontal arrows.*

Fig. S12. *Images of the same static air bubble in water acquired by (a) collimated (laser) and (b) diffuse (flashlamp) back illumination. Their corresponding image brightness profiles are presented in (c). (d) Comparison between the measured brightness profile [deducted from (b)] and the simulated irradiance profile.*

S3 Shockwave and bubble dynamics

Fig. S13. *Shockwave evolution over the edge of the stainless steel sample after laser pulse irradiation at l = 0.3 mm from the edge. Surrounding liquids are (a) water and (b) PEG. Pulse energy equals 10.6 mJ. White line in the inset image at 162 ns marks 100 μm.*

Fig. S14. *Shockwave front radius with respect to time after breakdown in water (black) and PEG (red). Dots represent experimentally determined values, while solid lines show the curves fitted by Eq. (S12).*

Fig. S15. *Shockwave propagation velocity with respect to time after breakdown in water (black) and PEG (red). Curves are derived from the fitted curves in Fig. S14 by using Eq. (S14).*

Fig. S16. *Bubble radius with respect to time after breakdown in water (black) and PEG (red). Dots represent experimentally determined values, while solid lines show the curves that are fitted by using Eq. (S15).*

Fig. S17. *Bubble wall velocity as a function of time after breakdown in water (black) and PEG (red).*

Fig. S18. *Pressure at the shockwave front and at the bubble wall as a function of time after breakdown in water (black) and PEG (red). Results for the bubble are calculated from the curves in Fig. S16 by using Eq. (S17).*

Fig. S19. *Pressure at the shockwave front and at the bubble wall as a function of radius in water (black) and PEG (red).*

Table S2. *Free fitting parameters obtained by fitting Eqs. (S12) and (S15) to the experimental measurements of shockwave and bubble radii.*

S4 Development of secondary cavity

Fig. S20. *Development of secondary cavity in water at 10 mJ pulse energy.*

Fig. S21. *Development of secondary cavity in water at 25 mJ pulse energy.*

Fig. S22. *Development of secondary cavity in water at 55 mJ pulse energy.*

Fig. S23. *Development of secondary cavity in ethanol at 10 mJ pulse energy.*

Fig. S24. *Development of secondary cavity in ethanol at 25 mJ pulse energy.*

Fig. S25. *Development of secondary cavity in ethanol at 55 mJ pulse energy.*

Fig. S26. *Development of secondary cavity in PEG at 10 mJ pulse energy.*

Fig. S27. *Development of secondary cavity in PEG at 25 mJ pulse energy.*

Fig. S28. *Development of secondary cavity in PEG at 55 mJ pulse energy.*

S5 Liquid injection into the cavitation bubble

Fig. S29. *Imaging of the liquid injection in ethanol from the side (same parameters as in Fig. 7c).*

Fig. S30. *Direct comparison of bubble dynamics induced in (a) water, (b) ethanol, and (c) PEG under same conditions. Image (c) is only illuminated from the back due to technical issues that could not be addressed in due time.*

Fig. S31. *Bubble dynamics of bubbles induced in (a) water (l = 0.2 mm) and (b) ethanol (l = 0.3 mm). Laser pulse energy equals 25 mJ.*

Fig. S32. *Asymmetrical re-entrant injection dynamics in a bubble induced at l = 1.1 mm in water. Laser pulse energy equals 25 mJ.*

Fig. S33. *Re-entrant injection dynamics in bubbles induced at l = 0.4 mm – 1.7 mm in (a-d) water and (e-h) ethanol. Laser pulse energy equals 25 mJ. Note the different time scale of (d) and (h) compared to others.*

Fig. S34. *Comparison of liquid injections in ethanol induced by laser pulses with pulse energies of (a) 10 mJ and (b) 25 mJ. Distance between the breakdown and the edge equals 0.3 mm.*

Fig. S35. *Comparison of liquid injections in ethanol for pulse energies of (a) 10 mJ, (b) 25 mJ, and (c) 55 mJ. The breakdown-edge distance equals 0.9 mm.*

Fig. S36. *Double breakdown in ethanol at pulse energy of 55 and l = 0.6 mm.*

S6 Influence of sample thickness

Fig. S37. *Comparison of the bubble dynamics in water at different thicknesses of the samples. Pulse energy equals 25 mJ. Breakdown is induced in the middle of the sample with thickness of (a) 25 μm, (b) 0.38 mm, (c) 1 mm, and (d) 2 mm.*

References

S1 Sample positioning and illumination

Observation of laser-induced cavitation bubble overflow over the edge was performed from two perspectives. In order to observe the dynamics that takes place inside the cavitation bubble, the process was observed *from the front* (Fig. S1a). In this case, the sample was clamped in an “H” configuration and the position of breakdown spot was coincident with the optical axis of the camera. In this configuration, the breakdown-edge distance l equals a.

Bubble expansion over the edge was monitored also *from the side* (Fig. S1b). Here, the breakdown spot was positioned out of the optical axis of the camera, which was collinear with the edge of the sample, as depicted in Fig. S1b. The sample was clamped in an “L” configuration. In this case, the breakdown-edge distance l equals b.

The focus of the image was set depending on desired observation. In case of the side-view, the object plane (i.e., the plane that appears sharp in the image) was set to coincide with the breakdown spot, as this is the plane that casts the shadow in our shadowgraphical experiment. In case of observation from the front, the surface of the sample that is nearer to the camera was set in focus, in order to observe the liquid injections that occur in this plane. Experiments with larger breakdown-edge distance in case of frontal observation therefore exhibit blurry bubble wall due to deliberate defocus (see Figs. S33d,h at 4 μs).

Dynamics of cavitation bubbles was captured by two different systems, explained in the main text. Illumination was utilized either by 30 ps laser pulses or by diffuse light source with pulse duration of ~1 ms. Temporal intensity profile of diffuse illumination source (flashlamp) that was used in experimental setup #1 is presented in Fig. S2.

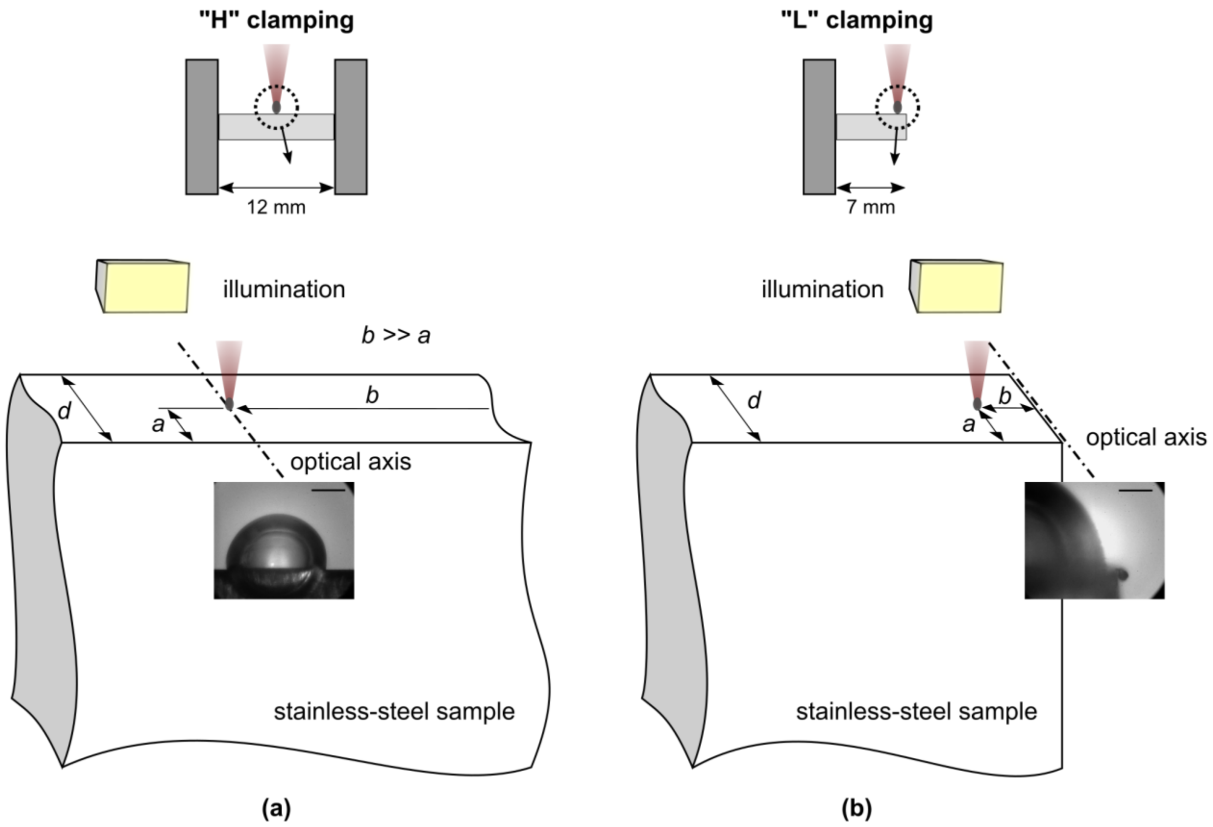

Fig. S1. Breakdown positioning in case of bubble overflow observation (a) from the front ("H" type clamping of the sample) or (b) from the side ("L" type clamping of the sample). The camera is located at the opposite side of the sample as illumination. Black lines in the corresponding acquired images represent 500 μm.

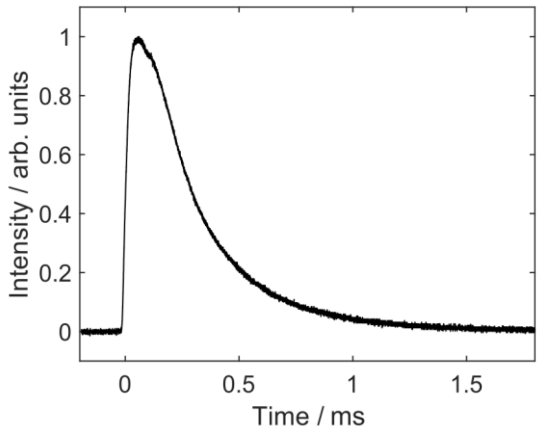

Fig. S2. Temporal intensity profile of diffuse illumination source (flashlamp) that is used in experimental setup #1.

S2 Ray tracing model

For clarifying the role of illumination diffusivity in bubble imaging, we developed a numerical model in Matlab. The model bases on laws of geometrical optics and simulates the trajectories of illuminating rays when passing through interfaces with different optical densities (i.e., bubble-liquid interfaces). The bubble of a radius R_b is assumed to contain vapor with refractive index $n_2 = 1$. Considered surrounding liquids are water and polyethylene glycol 300 (PEG) with refractive indices $n_1 = 1.33$ and $n_1 = 1.465$, respectively.

Rays in the simulation are characterized by the point of origin $O(x, y)$, normalized propagating vector $\vec{l}$ (pointing from the origin in direction of the ray propagation), relative intensity I, and refractive index of the medium the ray originates in. The geometry is defined in Cartesian coordinate system with bubble centered at the origin of the coordinate system, as shown in Fig. S3. Describing the ray by an equation of the geometrical line $y = kx + a$ and bubble wall as a circle with equation $x^2 + y^2 = R_b{}^2$, the points of intersection can be found from solutions of the quadratic formula

$$x_{1,2} = \frac{-B \pm \sqrt{B^2 - 4AC}}{2A} \quad , \tag{S1}$$

where coefficients A, B, and C are calculated as

$$A = k^2 + 1 \tag{S2}$$

$$B = 2ka \tag{S3}$$

$$C = a^2 - R_b^2 \tag{S4}$$

In general, this gives two solutions (intersection points), where the searched solution represents the point closer to the ray's origin $O(x, y)$.

Once the point of intersection with the bubble wall is determined, the angle of incidence in this point can be calculated. Depending on the ray direction that is described by the normalized vector $\vec{l}$ and the normal of the bubble wall described by the vector $\vec{n}$, the angle of incidence θ_i equals

$$\theta_\mathrm{i} = \cos^{-1}\left(-\vec{n}\cdot\vec{l}\right) \tag{S5}$$

where normal $\vec{n}$ is defined by

$$\vec{n} = \frac{(x,\ y)}{R_\mathrm{b}} \tag{S6}$$

and x, y in Eq. (S6) stand for the coordinates of the intersection point at the bubble wall. In this point, the ray can either be totally reflected or refracted depending on the angle of incidence, as depicted in Fig. S4.

In case of total reflection, the reflected ray retains the intensity of the incident ray, while the direction of travel is symmetric with respect to the normal $\vec{n}$. In case of light refraction, however, part of the ray is reflected with decreased intensity, while the remaining part is refracted inside the opposing medium. The angle of refraction θ_t is described by the Snell's law

$$\theta_\mathrm{t} = \sin^{-1}\left(\frac{n_1}{n_2}\sin\theta_\mathrm{i}\right). \tag{S7}$$

In case of light refraction, the amount of reflected light depends on the angle of incidence and the ratio of optical densities, as well as the direction of light polarization with respect to the plane of incidence. The reflectivity of s- and p-polarized light is described by Fresnel equations [S1]:

$$R_\mathrm{s} = \frac{\sin^2(\theta_\mathrm{i} - \theta_\mathrm{t})}{\sin^2(\theta_\mathrm{i} + \theta_\mathrm{t})} \tag{S8}$$

$$R_\mathrm{p} = \frac{\tan^2(\theta_\mathrm{i} - \theta_\mathrm{t})}{\tan^2(\theta_\mathrm{i} + \theta_\mathrm{t})}, \tag{S9}$$

in which indices s and p stand for the type of polarization. Here, s-polarization (called also TE) denotes that the electric field is oscillating perpendicular to the plane of incidence, while for p-polarization (called also TM) it oscillates parallel to the plane of incidence. The reflectivity of unpolarized light R_up can be calculated as the average reflectivity of both polarization types, yielding

$$R_{\mathrm{up}} = \frac{R_{\mathrm{s}} + R_{\mathrm{p}}}{2} \tag{S10}$$

Reflectivity of light for both types of polarization in dependence of the angle of incidence for cavitation bubble interface in water is presented in Fig. S5.

Following Eqs. (S7)-(S10) and characteristics of the light ray it is straightforward to define new rays with appropriate direction and intensity after the existing ray reaches the interface, as shown in Fig. S6. The process is then repeated for each newly determined ray as long as its intensity is above 1 % of the initial (illumination) ray.

After interacting with the bubble, (some of) the rays travel through the system of objective lens (the others are lost and do not reach the imaging plane). The objective is modeled with a thin lens and simulated by a ray transfer matrix. According to the objective focal length *f*, distance *y* and angle *θ* of the output rays with respect to the optical axis can be described by a system of linear equations as

$$\begin{pmatrix} y_2 \\ \theta_2 \end{pmatrix} = \begin{bmatrix} 1 & 0 \\ -\frac{1}{f} & 1 \end{bmatrix} \begin{pmatrix} y_1 \\ \theta_1 \end{pmatrix}, \tag{S11}$$

where indices 1 and 2 stand for input and output of the lens, respectively (Fig. S7).

The diameter of the lens *a* (representing also the aperture dimension) and its position with respect to the cavitation bubble x_{obj} were determined by measurements. Similarly, position x_{sen} and width of the sensor *w* were considered and incorporated into the model, presented in Fig. 2 of the main text. The line of pixels in the sensor is simulated as intervals with the size equal to physical pixel width. Numerical values of these parameters, which were considered in the simulations of diffuse and collimated illumination of experimental systems #1 and #2, are listed in Table S1. By counting the number of rays that intersect with each interval, relative irradiance profile is determined. Due to inconsistent number of rays and their density in the simulations of different illumination sources, the final irradiance profile is normalized to the average irradiance of the background without the bubble.

An example of ray tracing for a collimated illumination through a bubble wall (the red circle) with 50 initial rays is presented in Fig. S8. Both s- and p-polarization are considered in Figs. S8a and S8b, respectively. Typical rays that reach the sensor are labeled only in Fig. S8a. The black points indicate the origins of the starting rays. Brightness of the rays (in grayscale) is

proportional to their intensity, determined from the Fresnel relations [Eqs. (S8)–(S10)]. Black color indicates higher intensity. When simulating the irradiance profile (shown in Fig. 3 in the main text and in Figs. S9, S10, and S12), simulations were performed with a much higher number of initial rays (10^5–2×10^6), to minimize the effect of discretization.

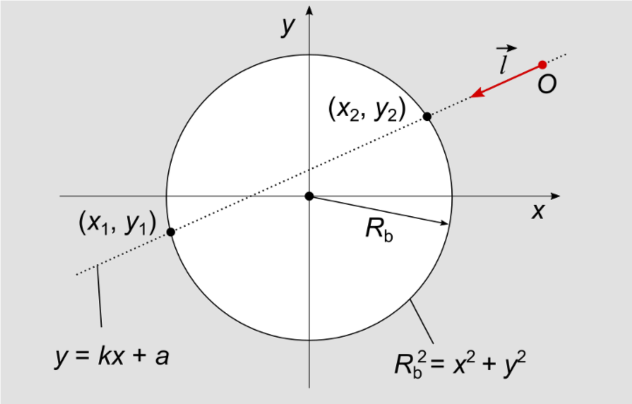

Fig. S3. Determining points of intersection with the bubble wall.

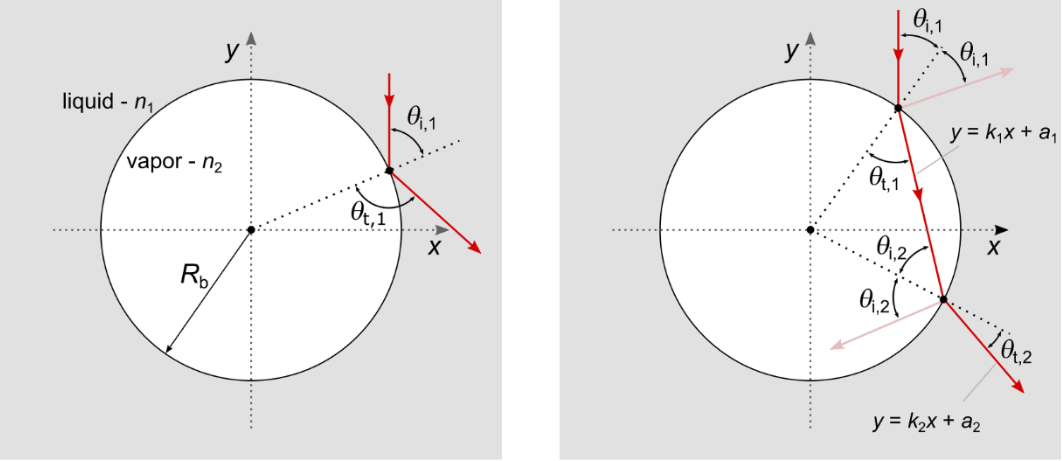

(a) (b)

Fig. S4. (a) Total reflection and (b) refraction of a light ray at the bubble wall.

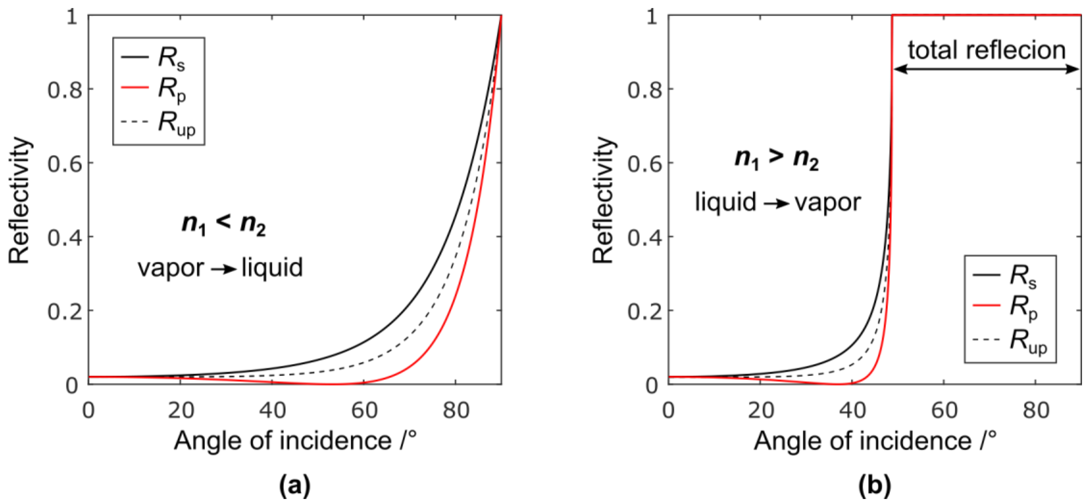

Fig. S5. Reflectivity of light for different angles of incidence when passing from (a) cavitation bubble (water vapor) into liquid (water) and (b) vice versa.

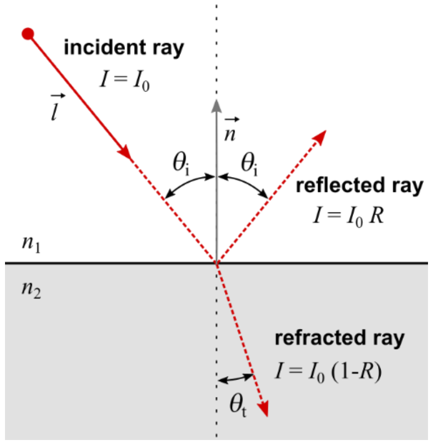

Fig. S6. Determining new rays after reaching the intersection point. Reflectivity R is determined from the Fresnel relations considering polarization orientation.

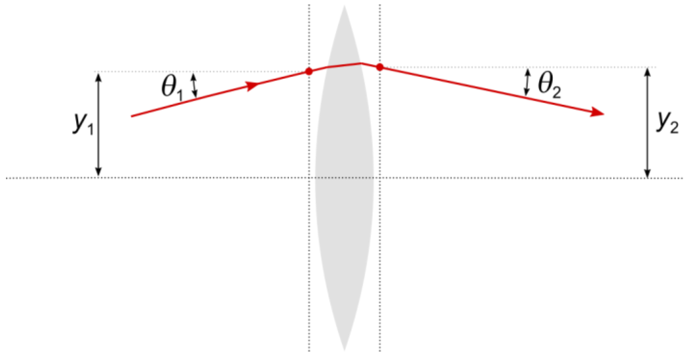

Fig. S7. Parameters in ray transfer matrix of objective lens.

Table S1. Parameters used in modelling of irradiance profiles for diffuse (experimental system #1, Fig. 3f in the main text) and collimated (experimental system #2, Fig. 3d in the main text) illumination of a vapor bubble with R_b = 0.7 mm.

Parameter	f /mm	L /mm	ΔL /µm	α /rad	$\Delta\alpha$ /mrad	x_{is} /mm
**Diffuse** (exp. sys. #1)	20	2.6	87	0.8	16	-0.8
**Collimated** (exp. sys. #2)	50	2.6	26	0	0	-0.8
**Parameter**	x_{obj} /mm	x_{sen} /mm	a /mm	w /mm	**pixel size** /µm	
**Diffuse** (exp. sys. #1)	22	242	11.2	20.8	66.5	
**Collimated** (exp. sys. #2)	67	264	11.2	6.1	4.8	

S2.1 Contribution of different ray types

Irradiance profile obtained by collimated unpolarized illumination is presented in Fig. S9, where the total irradiance (Fig. S9b) is decomposed to contributions of each type of rays #1-#4 (Figs. S9c-S9f). In addition to schematic presentation of ray types with different colors, Fig. S9a also shows their corresponding origin in terms of starting coordinate y with respect to the bubble radius R_b. The intervals of origins of different ray types are schematically presented in Fig. S9a by highlighted bands. Illuminating rays that originate outside of these intervals (indicated by vertical arrows in Fig. S9a) are either reflected (section between #2 and #3) or refracted (section between #1 and #2) out of the aperture of the objective lens and therefore do not reach the sensor. Clearly, the intervals of ray types are unique for individual optical setup due to different size proportions, apertures of the objective lens, as well as optical densities of the observed media. Nevertheless, considering our optical setup for collimated illumination (experimental setup #2) and assuming illumination of a vapor bubble with radius R_b = 1 mm in water, only the rays that originate from the following coordinates y reach the sensor: ($0 < y < 0.12R_b$) for rays #1, ($0.69R_b < y < 0.75R_b$) for rays #2, ($0.999R_b < y < R_b$) for rays #3, and ($R_b < y$) for rays #4.

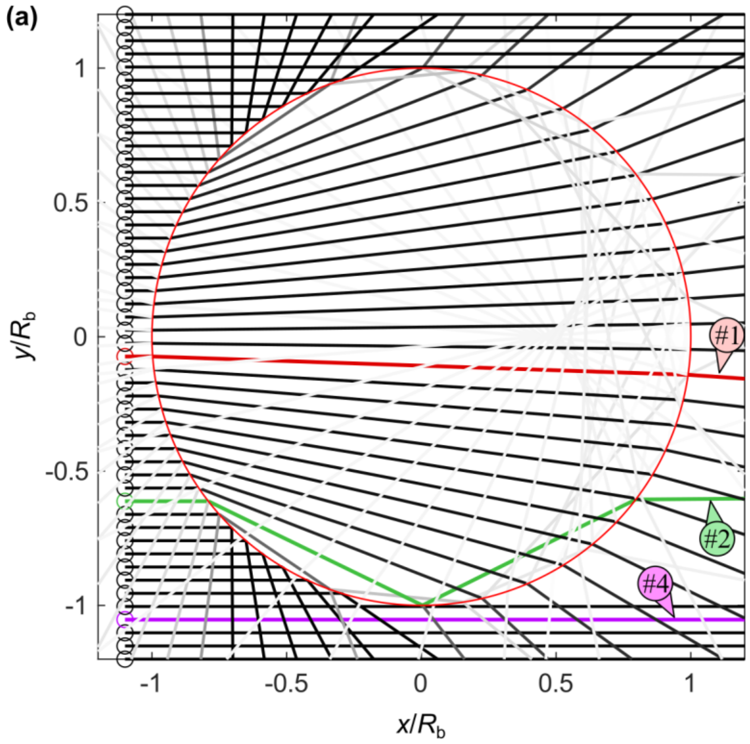

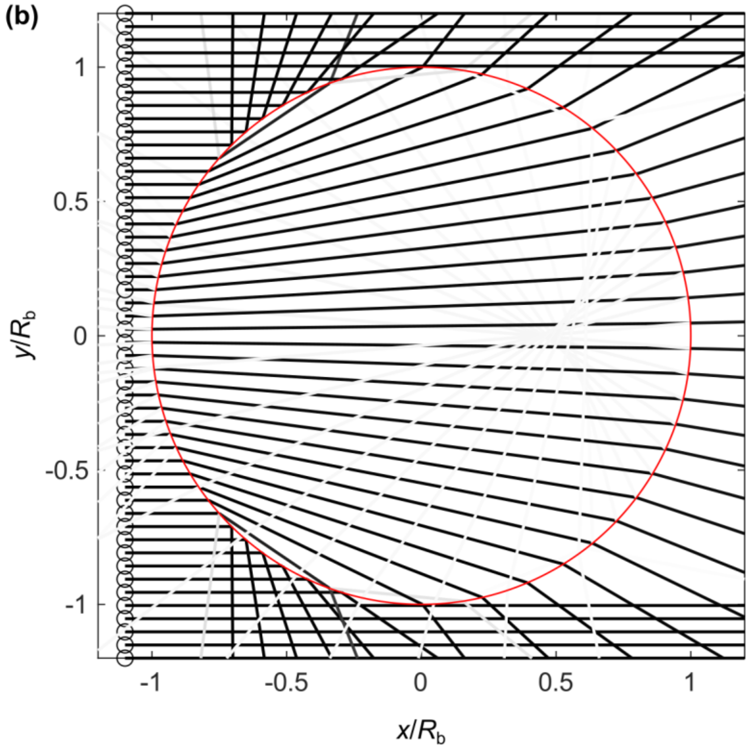

Fig. S8. Ray tracing simulations of collimated illumination with 50 parallel initial rays. (a) s- and (b) p-polarizations are considered. Brightness of the lines corresponds to relative intensity of the ray, ranging from 0 (white) to 1 (black). Characteristic rays of types #1, #2, and #4 are presented in (a) with red, green, and pink color, respectively.

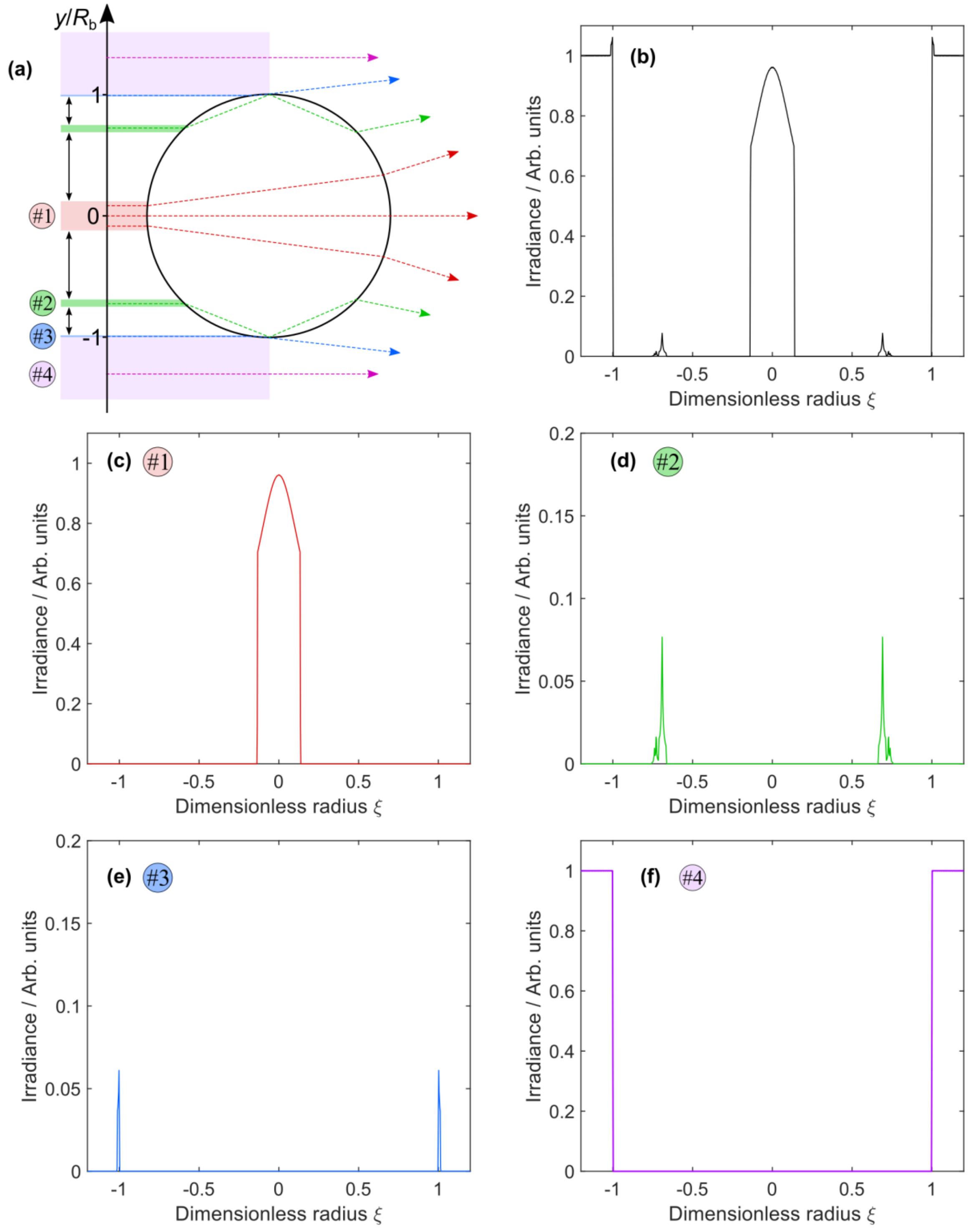

Fig. S9. (a) Sections (intervals) of collimated illumination source that contribute (by superposition) to (b) the total irradiance profile of the vapor bubble in water. Individual contribution of ray types #1-#4 is shown in (c)-(f), respectively.

S2.2 Influence of light polarization

The effect of light polarization is most noticeable in the bright ring that appears due to light reflections inside the bubble (type #2 rays; Fig. S9d). The rays that contribute to formation of the ring are those that reflect inside the bubble multiple times before exiting. Therefore, reflectivity plays an important role in determining the brightness of the perceived ring (shown in Fig. S5a). Considering Fresnel relations [Eqs. (S8)–(S9)], higher reflectivity of the s-polarized light at the bubble wall yields higher intensity of the light "transmitted" through the bubble by reflections, which can be detected as increased brightness of the ring. In case of linearly polarized illumination of a spherical bubble, the brightness of the ring should therefore not be equal all around the ring, but rather symmetrical with respect to polarization. In order to prove this experimentally, a static air bubble was appended to the surface of the sample, held together merely by surface tension. The same bubble was further illuminated with different illumination conditions, making results directly comparable.

The effect of rotating the direction of the linear polarization of illumination light is shown in Fig. S10. Here, the static bubble is illuminated with horizontally (Fig. S10a) and vertically (Fig. S10b) oriented polarizations. Albeit the illumination intensity is not equal in both images, the image brightness profiles were deducted from the acquired images at the location marked by horizontal arrows in Figs. S10a and S10b. It can be seen from corresponding image brightness profiles in Figs. S10e and S10f, that the bright ring (resulting from reflections inside the bubble, marked by vertical arrows at $|\xi|\sim 0.7$) is brighter in case of vertical polarization, which presents s-polarization with respect to the horizontal plane (plane of image brightness profile). Should we deduct the image brightness profile in the vertical direction, very similar results would be obtained, however this time the brighter ring would be detected with horizontal polarization, since horizontal polarization would now represent s-polarization. The latter can be easily distinguished from acquired images with increased brightness and contrast, where the ring in Fig. S10c appears brightest at the top, while in Fig. S10d it is noticeably brighter at the sides. The obtained results also agree with simulated irradiance profiles in Figs. S10g and S10h.

Irradiance of the central part of the bubble is fairly unaffected by direction of polarization due to low angle of incidence of (type #1) illuminating rays at the bubble interface, which results in practically same reflectivity of both types of polarization (Fig. S5, angles of incidence <20°).

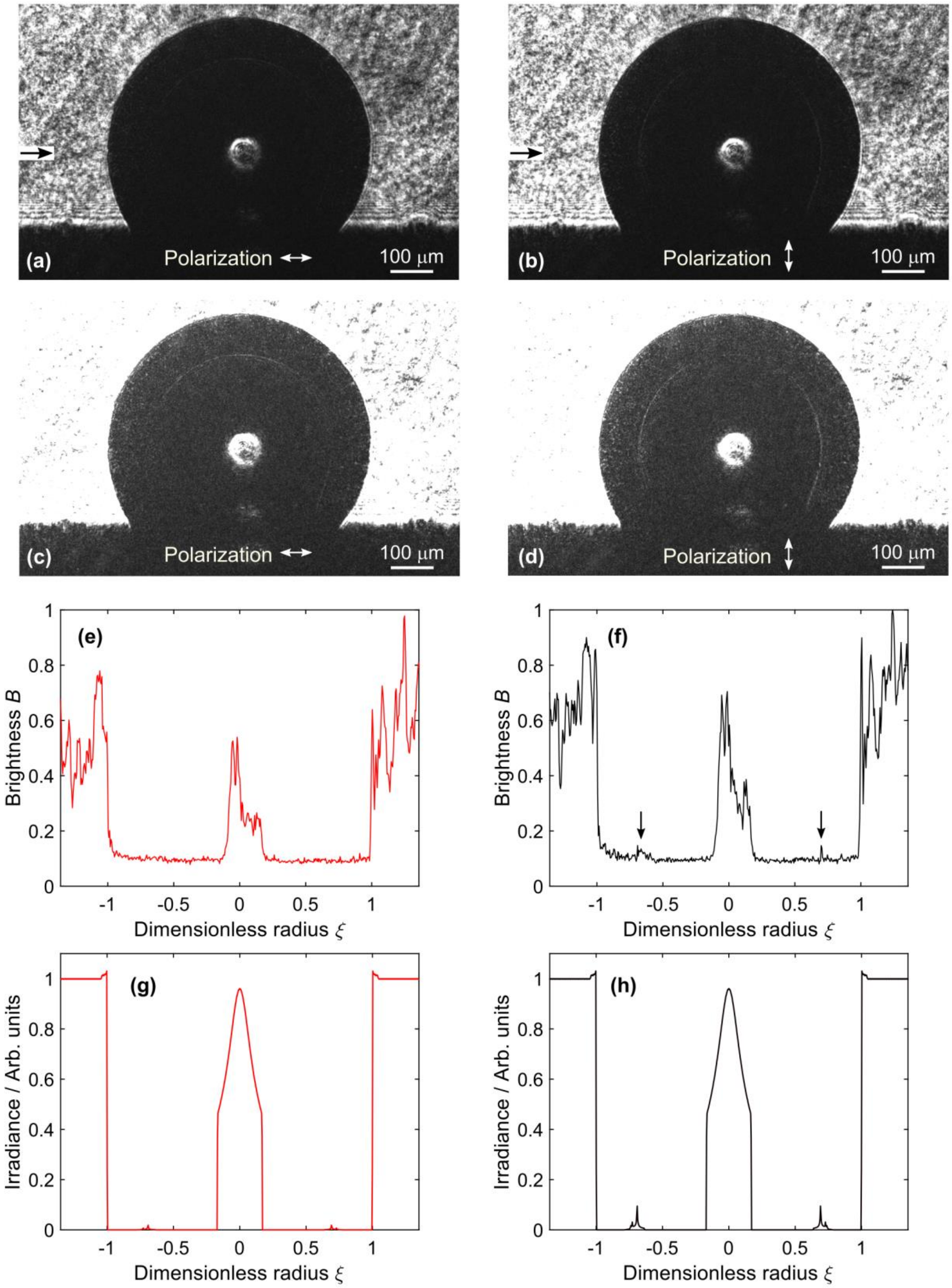

Fig. S10. Images of the *same* static air bubble acquired by (a) p-polarized and (b) s-polarized collimated (laser) back illumination (experimental setup #2). Brightness and contrast of the image are significantly increased in (c) and (d), respectively, for better visibility of the bright ring. Image brightness profiles, deducted from (a) and (b) are shown in (e) and (f), while simulated irradiance profiles are presented in (g) and (h), respectively.

S2.3 Influence of angle of incidence

Similar to previous experiments with different polarizations, a static air bubble appended to the sample surface was observed with different illuminating conditions to enable direct comparison of the results. Figure S11 shows the effect of illuminating a bubble with a collimated light source incident at 0° (Fig. S11b) and 45° (Fig. S11c) with respect to the optical axis. As visible from Fig. S11c, illuminating by an angle of 45° (from the left) leads to formation of only two bright spots in the image sensor. The outer spot (in this case the left one) is caused by total reflection of light rays at the outer bubble wall (ray type #3), as shown in the ray tracing simulation in Fig. S11a. The position of the peak of this bright spot with respect to the bubble wall depends on the angle of incidence – higher angle yields formation of the spot closer to the bubble center. When diffuse illumination source is used for illuminating the bubble, these reflections were shown [S2] to cause a sizing error of up to 30 % for an infinitely large plane source (with emitting angle $\alpha = 180°$). Considering our optical setup for collimated illumination (experimental setup #2) and illumination from 45°, the peak of this "wall reflection" spot is detected at $\xi \sim -0.92$, as seen in Fig. S11d. Should the illumination be incident at the same angle from the right instead of from the left, the result would be equal but mirrored over the vertical axis.

The second bright spot, closer to the center of the bubble (peak at $\xi \sim -0.58$), results from rays that travel through the bubble by either only refraction (type #1) or also reflection at the inner bubble wall (type #2), as shown in Fig. S11a. Compared to rays reflected off the outer bubble wall (type #3), these travel through the bubble and thus increase the illuminated area inside the bubble, broadening the view of phenomena occurring inside. Type #4 rays in the meantime travel out of the aperture of objective due to their large angle of incidence – the background is dark.

A diffuse illumination source can also be considered as a sum of many collimated sources that are incident at different angles, which was also implemented in our ray tracing model. Figure S12 shows experimental comparison of illuminating the same static air bubble with a collimated (i.e., laser) source (Fig. S12a) and a diffuse (i.e., flashlamp) source (Fig. S12b). Deducted image brightness profiles in Fig. S12c confirm the difference in irradiance gradient at the bubble wall ($|\xi| \sim 1$). It is clearly seen that the edge of the bubble in case of diffuse illumination becomes blurred due to contribution of direct (type #4) rays that travel beside the bubble wall at an angle, as well as reflections off the outer bubble wall (type #3), as explained in Fig. S11d. By

measuring only the apparent (darkened) part of the bubble, we estimate up to around 5% error in size determination can be made (with common optical setups, such as our experimental setup #1) due to wrong interpretation of the irradiance profile.

Experiments agree well with the simulated results. Figure S12d shows the simulated irradiance profile compared to the measured one with reduction of background noise (zero irradiance is assumed at brightness level that equals to 0.2). The emitting angle of illumination source considered in the simulation is assumed to equal 0.4 rad with respect to the optical axis. The most significant deviation of the simulated irradiance profile from the experimentally determined one is in the intensity profile of the central irradiated part of the bubble (rays #1). The simulated irradiance in this part exhibits a fairly constant value, while the experimental values show that the intensity decreases from the bubble’s center outward. The deviation can be explained by angular intensity distribution of the illumination source, which determines the intensity of rays that are incident at different angles. Since the widening of the central illuminated area in the bubble can be attributed mostly to type #1 rays that are incident at higher angles, the irradiance in this part depends on their intensity. The angular intensity distribution of the source is assumed a “pillbox” distribution in the simulations, meaning constant intensity through all angles of incidence. True angular intensity distribution of the flashlamp illumination used in the experiments, however, most likely decreases with the angle. This leads to a more noticeable decrease in irradiance (at roughly $0.15 < |\xi| < 0.5$ in Fig. S12d) compared to the simulation.

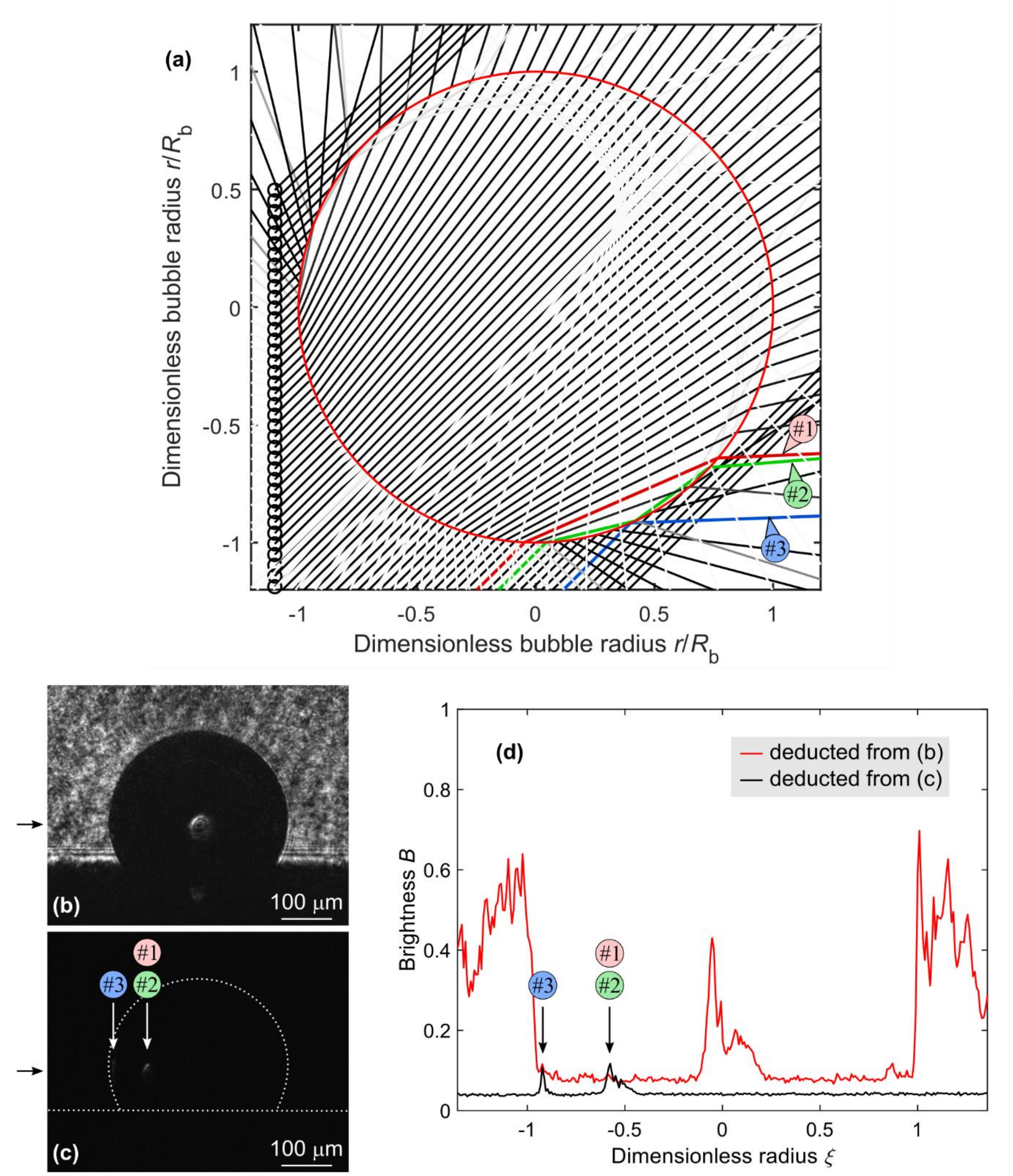

Fig. S11. (a) Ray tracing simulation of collimated illumination incident at 45° (with respect to the horizontal optical axis). Images of the *same* static air bubble are acquired by collimated back illumination with the angle of incidence equal to (b) 0° and (c) 45° (from the left) with respect to the optical axis. (d) Image brightness profile deducted from (b) and (c) at the locations marked by horizontal arrows.

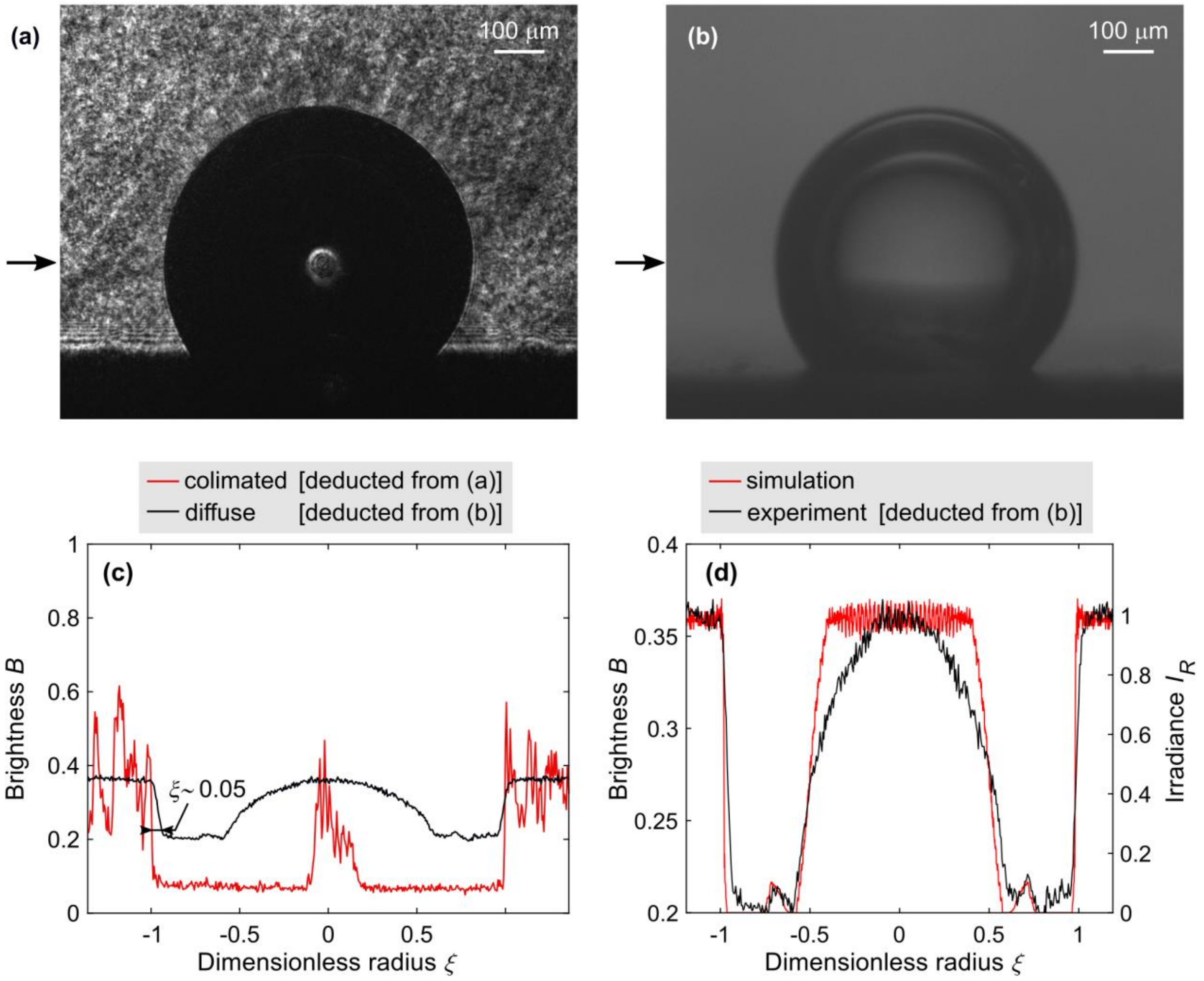

Fig. S12. Images of the *same* static air bubble in water acquired by (a) collimated (laser) and (b) diffuse (flashlamp) back illumination. Their corresponding image brightness profiles are presented in (c). (d) Comparison between the measured brightness profile [deducted from (b)] and the simulated irradiance profile.

S3 Shockwave and bubble dynamics

In order to characterize and evaluate the dynamics occurring within the first few microseconds after the excitation, radii of the shockwave and cavitation bubble were measured from the images (Fig. S13) acquired by experimental setup #2 with great temporal resolution. Multiple breakdown events were captured at different time instances after the excitation pulse to capture a temporal evolution of the laser-induced phenomena. Experiments were performed in water (Fig. S13a) and PEG (Fig. S13b).

A sudden release of energy after an excitation-laser pulse is absorbed in the metal and leads to formation of plasma and, consequently, to explosive expansion of the surrounding medium. This results in *(i)* shockwave formation, which ultrasonically propagates into the liquid outward from the breakdown position and *(ii)* development of a cavitation bubble.

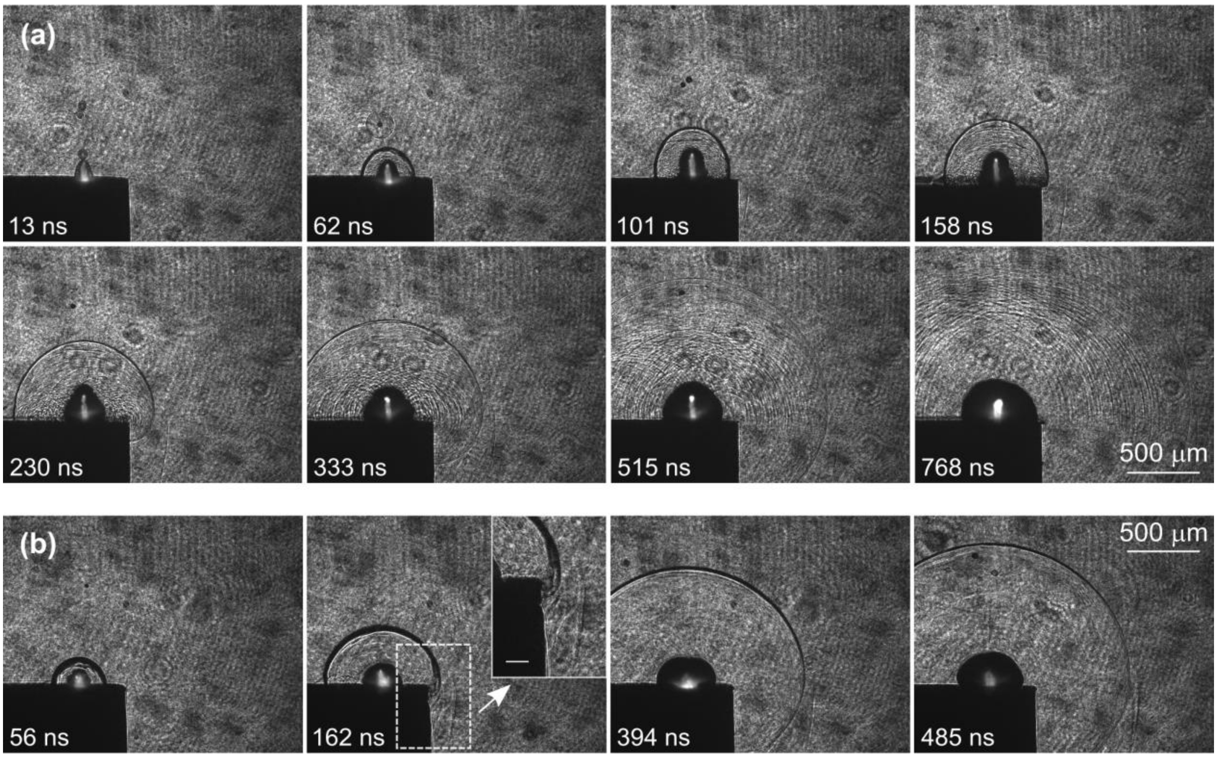

Fig. S13. Shockwave evolution over the edge of the stainless steel sample after laser pulse irradiation at l = 0.3 mm from the edge. Surrounding liquids are (a) water and (b) PEG. Pulse energy equals 10.6 mJ. White line in the inset image at 162 ns marks 100 μm.

S3.1 Evaluation of shockwave dynamics

The shockwave radius R_s as a function of time t after the excitation-laser pulse was measured by fitting a circle to the shock wave front that is visible as a thin dark ring in Fig. S13. Slight deviations of the pulse-to-pulse repeatability due to pulse energy variation and/or variation in the optical breakdown do not allow to calculate shockwave velocity by direct derivation of the measured radii that are shown by the dots in Fig. S14. To make this possible, the measured radii were fitted by using the following curve:

$$R_s = R_0\left[n^{\frac{n}{1-n}}\left(\left(n^{\frac{-1}{1-n}}\left(\frac{c_0}{R_0}t\right)+1\right)^n-1\right)\right]^{1/n}, \qquad \text{(S12)}$$

where the characteristic radius R_0, the speed of sound c_0, and n, are free fitting parameters. The fitting curve given by Eq. (S12) is derived from the Jones's [S3] generalized trajectory for blast waves with proper limit for the strong blast waves, when $t \to 0$, $R_s \to \left(R_0^{\frac{n-1}{n}} c_0^{\frac{1}{2}} t\right)^{1/n} \propto t^{1/n}$ as well as for the acoustic waves, i.e., when $t \to \infty$, $R_s \to c_0 t$. The fitted curves [Eq. (S12)] are shown by the solid lines in Fig. S14. The fitted free parameters are listed in Table S2.

The goodness of fit was evaluated as the average relative difference between the measured and fitted value through the following equation:

$$\sigma = \frac{1}{N}\sqrt{\sum_{i=1}^{i=N}\left(\frac{\Delta R_i}{R_{i,\mathrm{fit}}}\right)^2}, \qquad \text{(S13)}$$

where ΔR_i stands for the radius difference between the i-th measurement (point) and the fitted radius $R_{i,\mathrm{fit}}$ at the same time and N equals the number of the all measured points. For shockwave radius in water and PEG, this average relative difference was estimated to ± 1% and ± 0.9%, respectively.

From the temporal evolution of the shockwave radius, its velocity u_s can be determined simply by deriving

$$u_s = \frac{dR_s}{dt} \ . \tag{S14}$$

Results for the shockwave velocity in water and PEG are presented in Fig. S15.

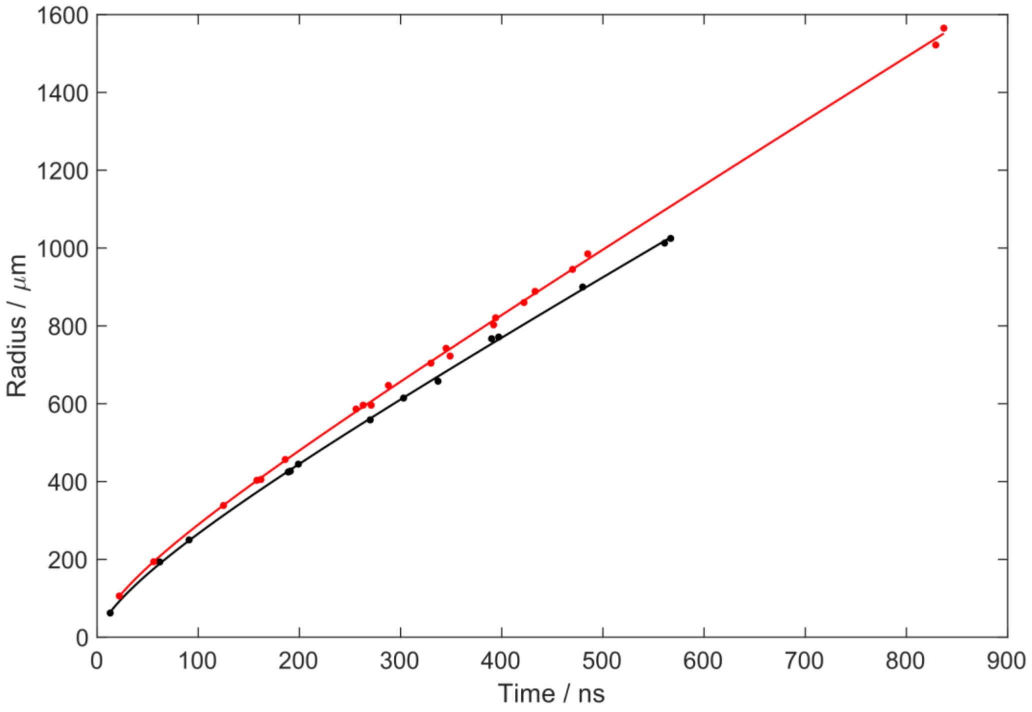

Fig. S14. Shockwave front radius with respect to time after breakdown in water (black) and PEG (red). Dots represent experimentally determined values, while solid lines show the curves fitted by Eq. (S12).

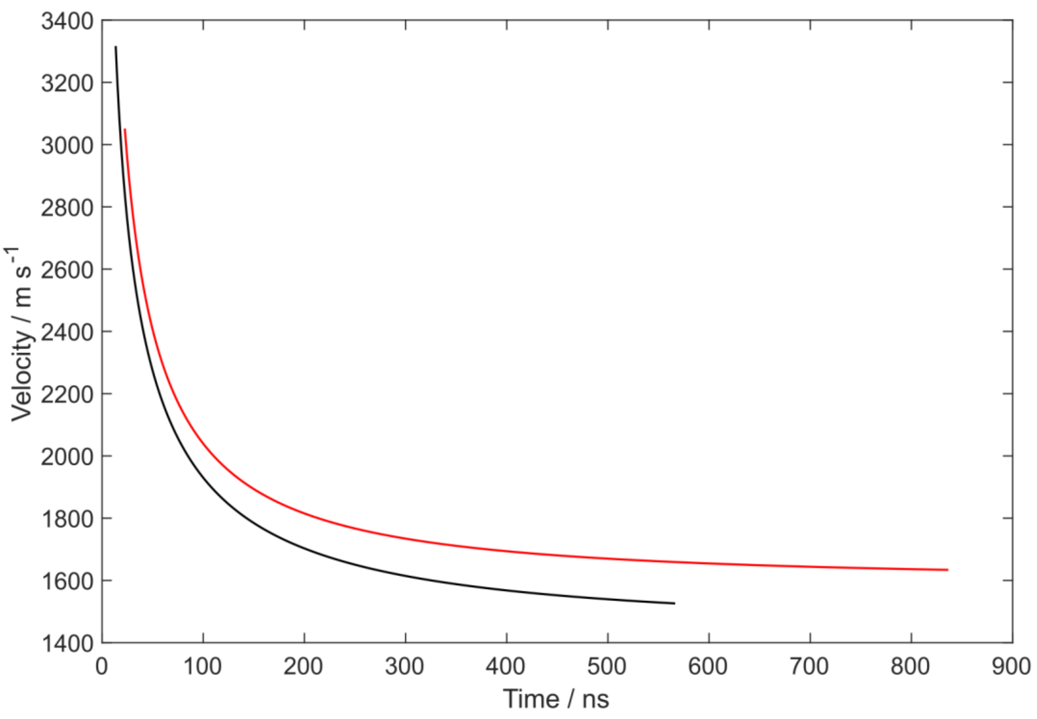

Fig. S15. Shockwave propagation velocity with respect to time after breakdown in water (black) and PEG (red). Curves are derived from the fitted curves in Fig. S14 by using Eq. (S14).

S3.2 Evaluation of bubble dynamics

With the experimental setup #2, we captured single images of bubbles from single excitation-laser pulses. In this way, we measured the radii of *individual* (different) bubbles as a function of time (the dots in Fig. S16). The first and second temporal derivative of radius as a function of time are needed to calculate the pressure difference (see Eq. (3) in the main text). Thus, we fitted the following curve (solid lines in Fig. S16) to the measured data:

$$R_b(t) = r_0 \left(\frac{t}{t_0} \right)^n . \quad \text{(S15)}$$

Fitting parameters r_0, t_0, and n are listed in Table S2. The average relative difference between the measured bubble radius and its fitted value at the corresponding time [Eq. (S13)] was estimated to ± 3% and ± 2% in water and PEG, respectively. To calculate the pressure difference at the bubble wall, Δp, first and second derivative of the fitting curve [Eq. (S15)] should be calculated as

$$\dot{R}_{\mathrm{b}}(t) = \frac{r_0 n}{t_0^n} t^{n-1}$$
$$\ddot{R}_{\mathrm{b}}(t) = \frac{r_0 (n^2 - n)}{t_0^n} t^{n-2}. \quad \text{(S16)}$$

Thereby, the pressure difference can be estimated from the fitted data as:

$$\Delta p = \rho_0 \frac{r_0^2 n^2}{t_0^{2n}} \left(\frac{5}{2} - \frac{1}{n} \right) t^{2n-2} \quad \text{(S17)}$$

Figure S17 shows the velocity of the bubble wall as a function of time, calculated from the first equation in Eq. (S16). Pressure as a function of time is shown in Fig. S18, while Fig. S19 shows the pressure as a function of radius.

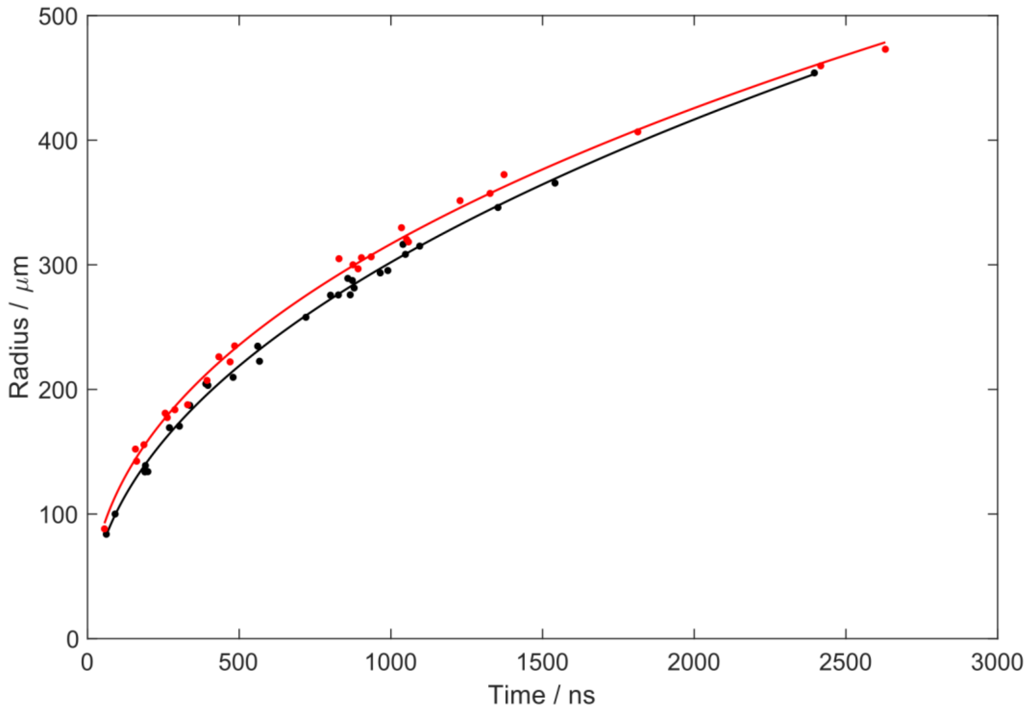

Fig. S16. Bubble radius with respect to time after breakdown in water (black) and PEG (red). Dots represent experimentally determined values, while solid lines show the curves that are fitted by using Eq. (S15).

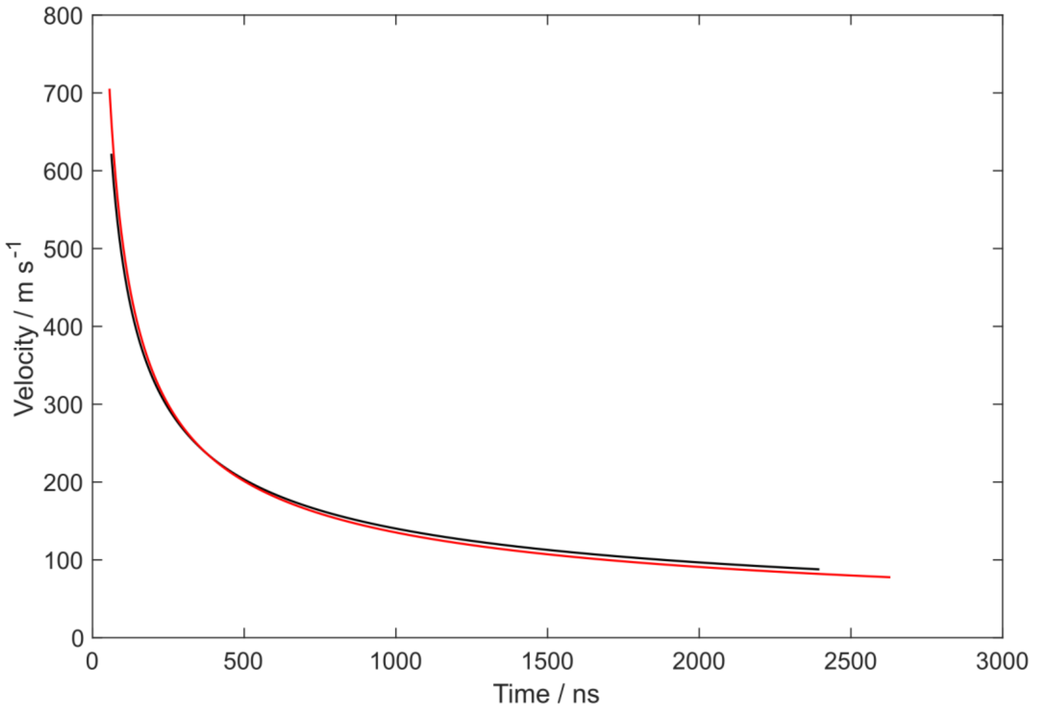

Fig. S17. Bubble wall velocity as a function of time after breakdown in water (black) and PEG (red).

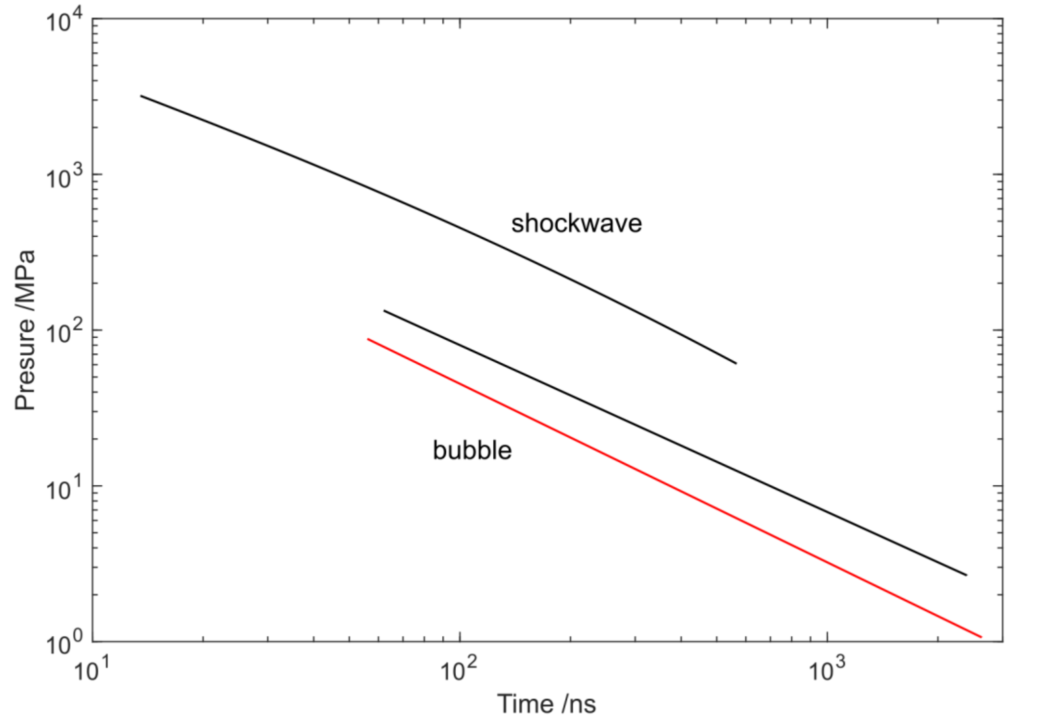

Fig. S18. Pressure at the shockwave front and at the bubble wall as a function of time after breakdown in water (black) and PEG (red).

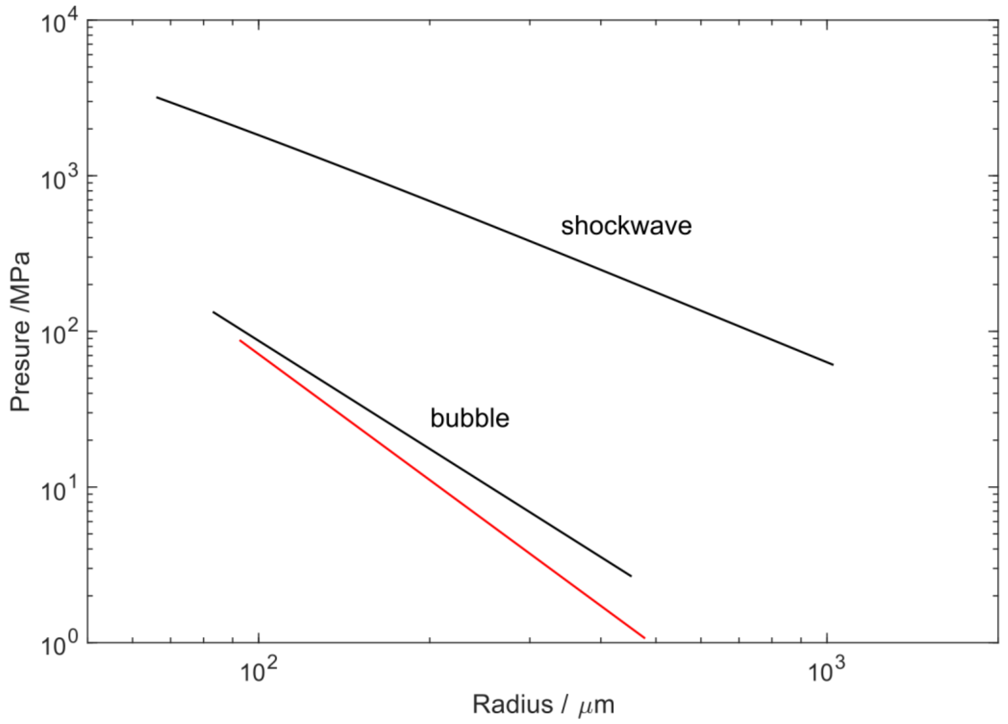

Fig. S19. Pressure at the shockwave front and at the bubble wall as a function of radius in water (black) and PEG (red).

Table S2. Free fitting parameters obtained by fitting Eqs. (S12) and (S15) to the experimental measurements of shockwave and bubble radii.

**Liquid**	**Shockwave – Eq. (S12)**			**Bubble – Eq. (S15)**		
	$\boldsymbol{R_0}$ /μm	$\boldsymbol{c_0}$ /km s^{-1}	$\boldsymbol{n}$	$\boldsymbol{r_0}$ /μm	$\boldsymbol{t_0}$ /ns	$\boldsymbol{n}$
Water	752.6	1.44	0.99	9.75	0.62	0.46
PEG	592.2	1.60	1.22	14.77	0.76	0.43

S4 Development of secondary cavity

In this section, we present the side observation of dynamics occurring ±4 μs after formation (inception) of the secondary cavity. The point of inception was determined manually by finding the first frame within video with a visible secondary cavity. In Figs. S20-S28, this frame is shown in the third consecutive image of each row. The number on the top of these images denotes the liquid flow velocity over the edge at this instance. Time in the left bottom corner denotes time after breakdown. Values on the left of each row represent breakdown-edge distance *l*.

S4.1 Water

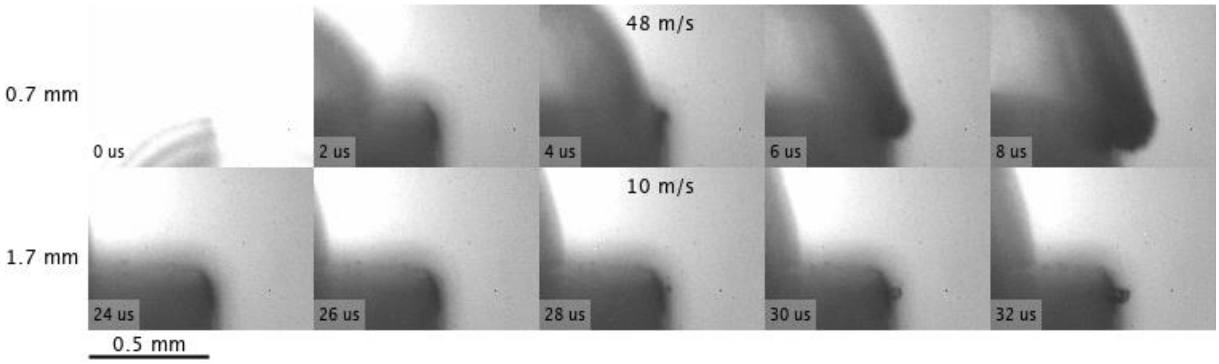

Fig. S20. Development of secondary cavity in water at 10 mJ pulse energy.

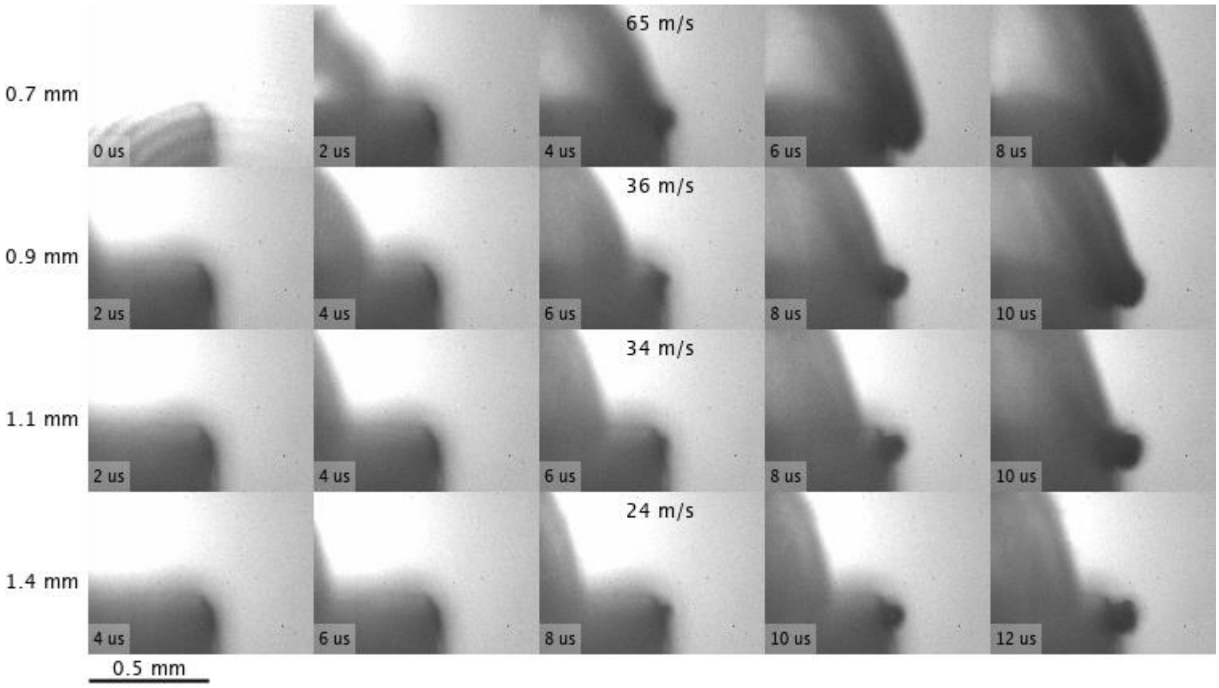

Fig. S21. Development of secondary cavity in water at 25 mJ pulse energy.

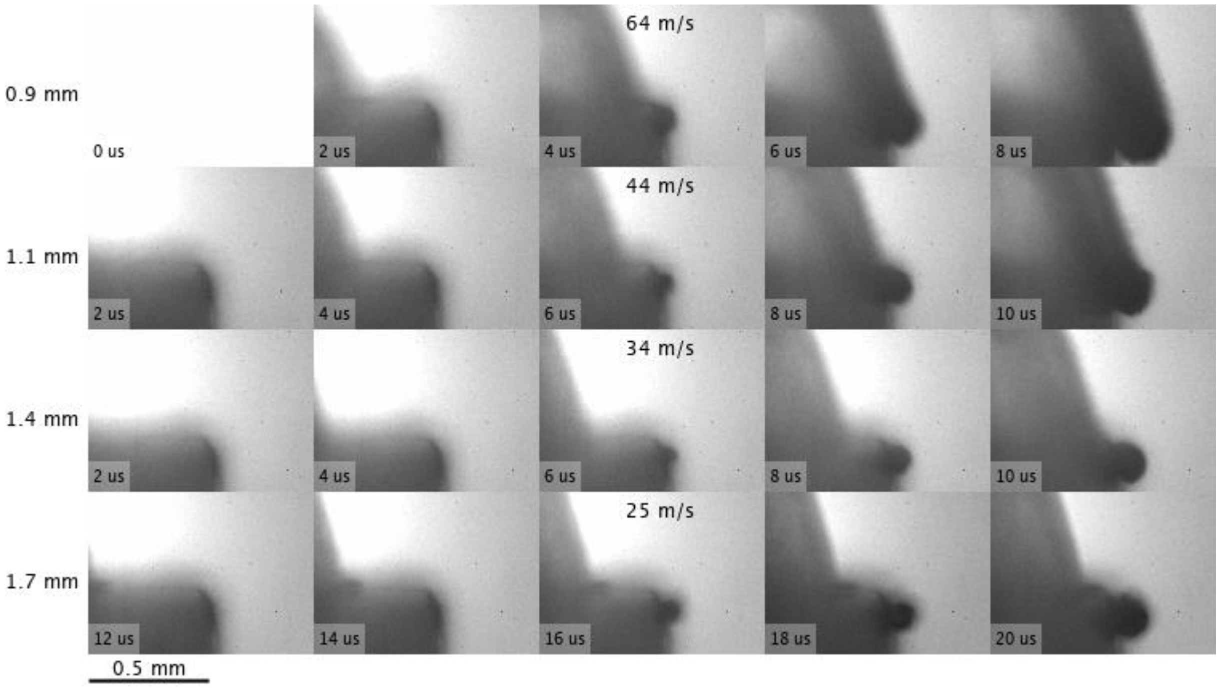

Fig. S22. Development of secondary cavity in water at 55 mJ pulse energy.

S4.2 Ethanol

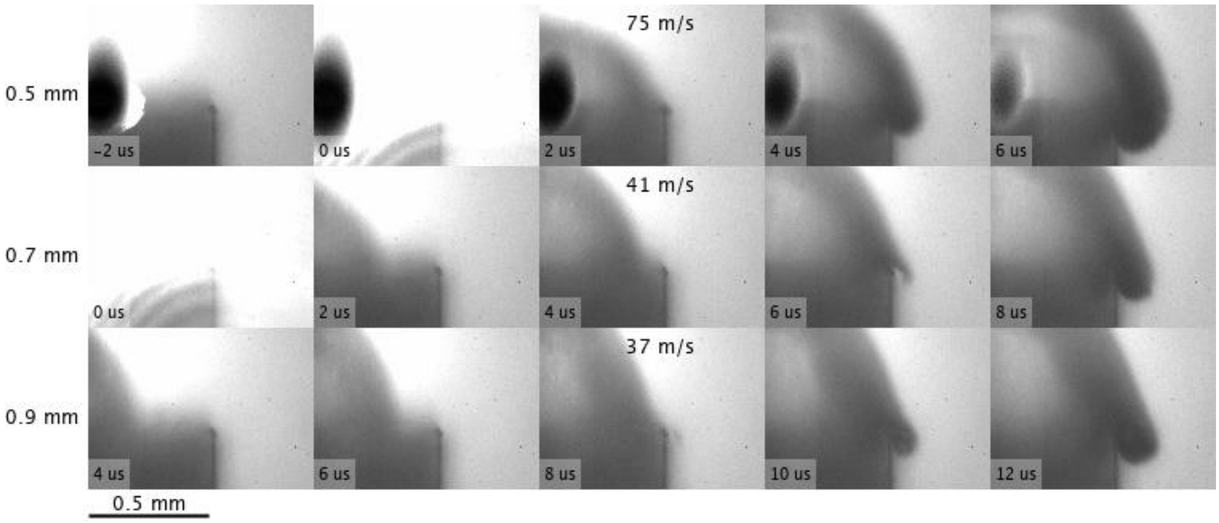

Fig. S23. Development of secondary cavity in ethanol at 10 mJ pulse energy.

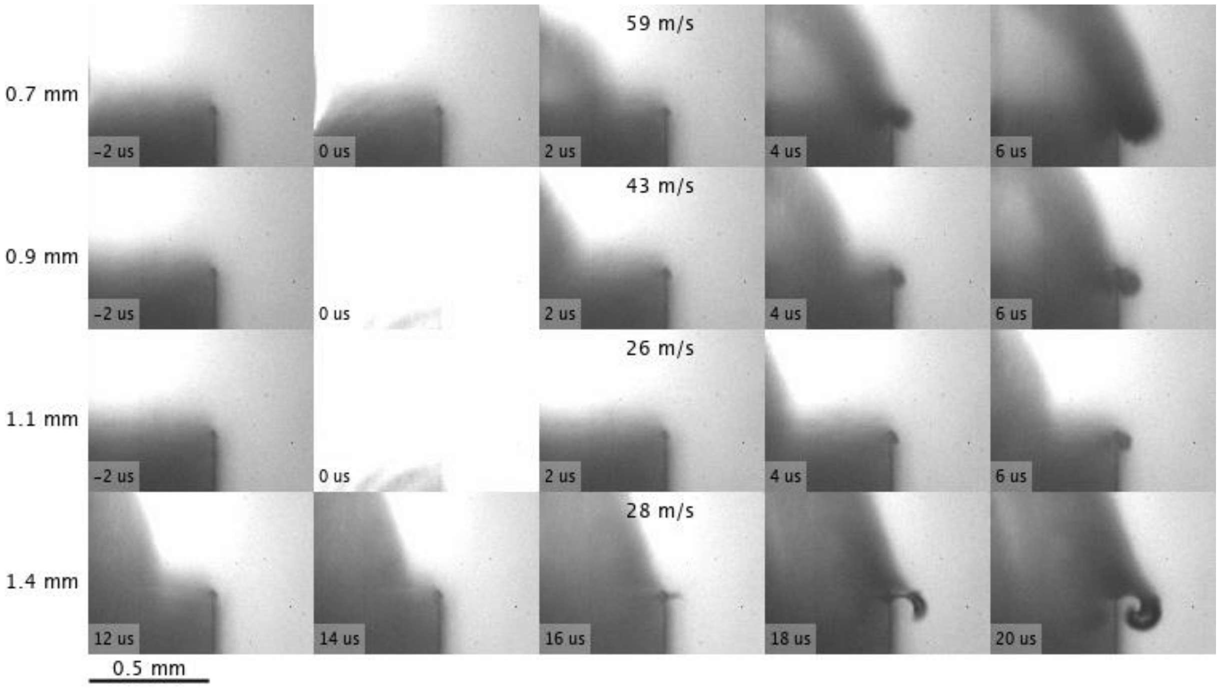

Fig. S24. Development of secondary cavity in ethanol at 25 mJ pulse energy.

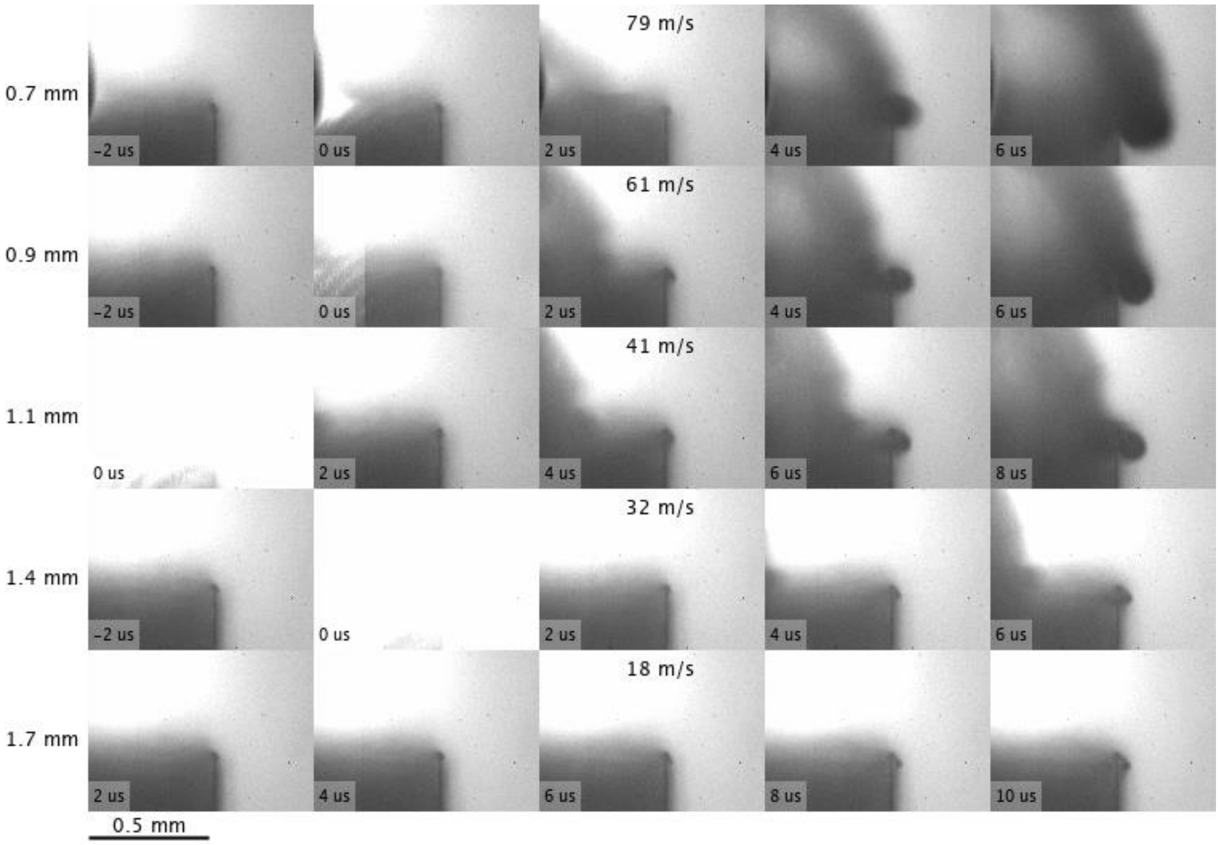

Fig. S25. Development of secondary cavity in ethanol at 55 mJ pulse energy.

S4.3 Polyethylene glycol

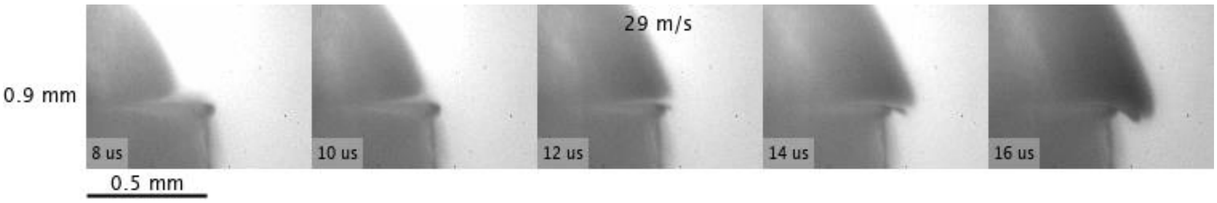

Fig. S26. Development of secondary cavity in PEG at 10 mJ pulse energy.

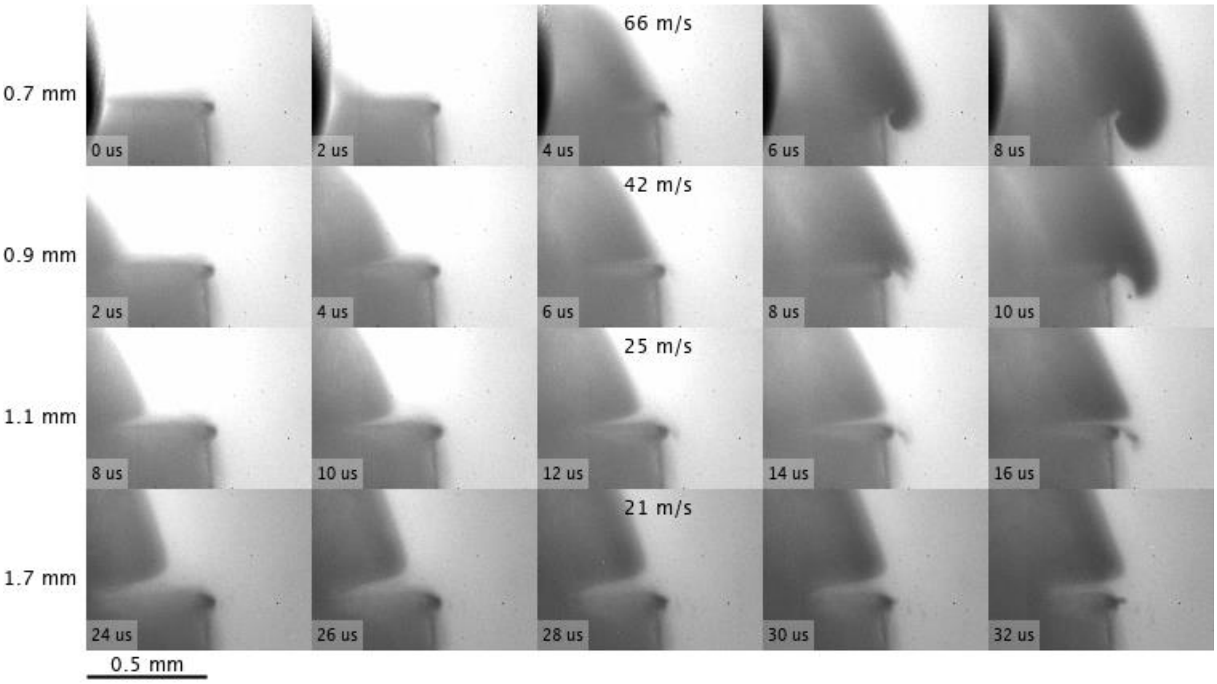

Fig. S27. Development of secondary cavity in PEG at 25 mJ pulse energy.

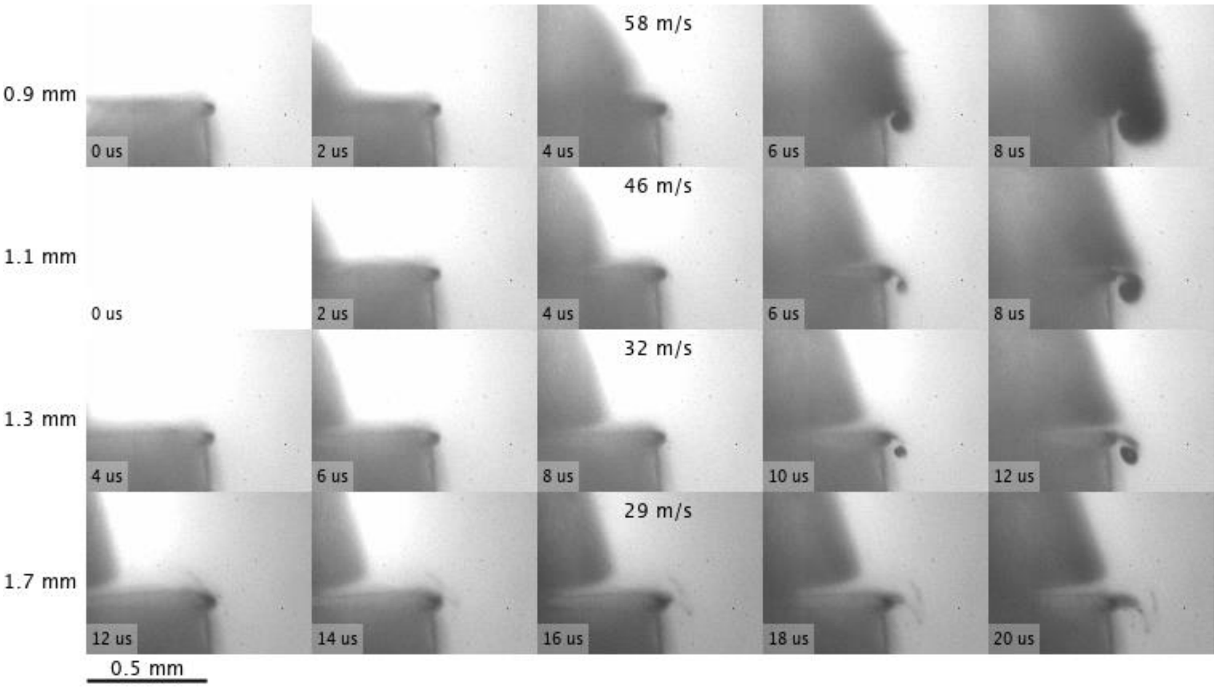

Fig. S28. Development of secondary cavity in PEG at 55 mJ pulse energy.

S5 Liquid injection into the cavitation bubble

This section shows the dynamics of laser-induced cavitation bubbles from either the front or the side, captured with an ultrafast camera (experimental setup #1). Images were captured at 500 kfps. Thus, the time between two consecutive frames equals 2 μs. The image size equals 2.09×1.74 mm^2.

S5.1 Observation from the side

Figure S29 shows the liquid injection in ethanol observed from the side, induced under the same conditions as those in Fig. 7c, i.e., l = 0.9 mm and 25 mJ pulse energy. Injection can be seen propagating at a slight angle with respect to the vertical sample surface. Injections that can be seen left of the marked injection in Fig. S29 at 120 μs result from the same phenomena that occur at the edge (horizontal dashed line of the sample mark) of the sample surface facing the ultrafast camera.

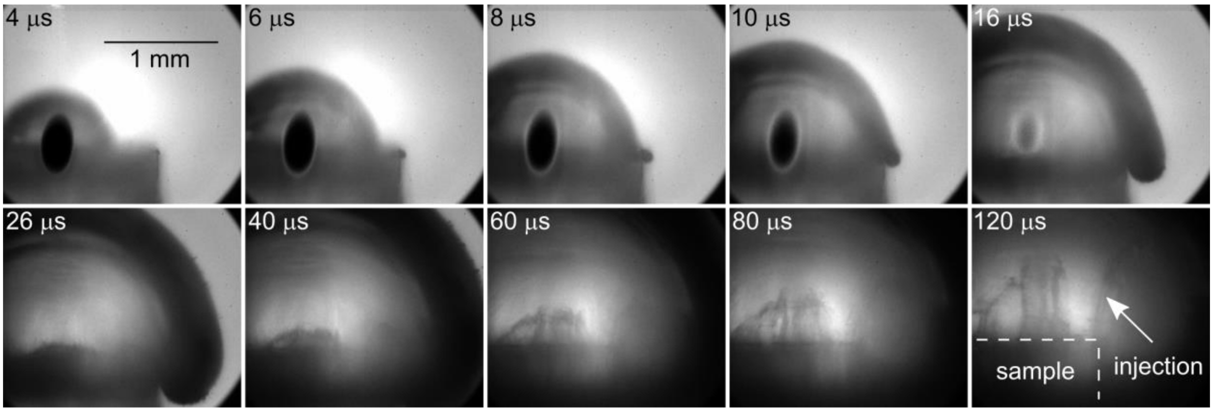

Fig. S29. Imaging of the liquid injection in ethanol from the side (same parameters as in Fig. 7c).

S5.2 Influence of surrounding liquid

Figure S30 shows comparison of bubble dynamics induced in water, ethanol, and PEG. Pulse energy equals 25 mJ, while l = 0.9 mm.

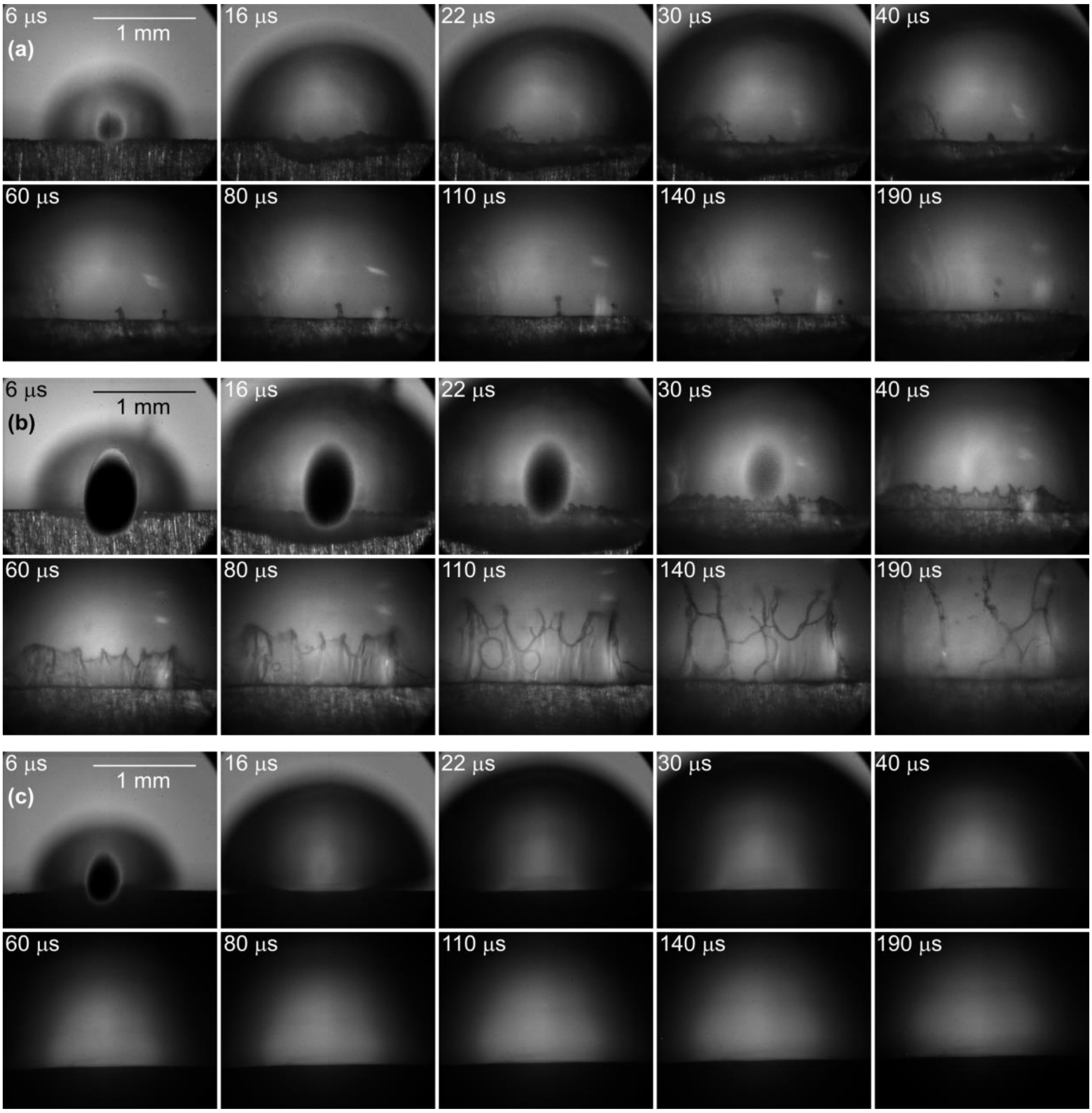

Fig. S30. Direct comparison of bubble dynamics induced in (a) water, (b) ethanol, and (c) PEG under same conditions. Image (c) is only illuminated from the back due to technical issues that could not be addressed in due time.

S5.3 Influence of breakdown-edge distance

Figure S31 shows the case when the distance between the breakdown and the edge is insufficient for visible liquid injection in water and ethanol.

Figure S32 shows an asymmetrical re-entrant injection that occurred at l = 1.1 mm in water at 25 mJ pulse energy.

Figure S33 supports Fig. 8 in the main paper. Full temporal evolution of each individual injection from the inset images in Fig. 8 is presented.

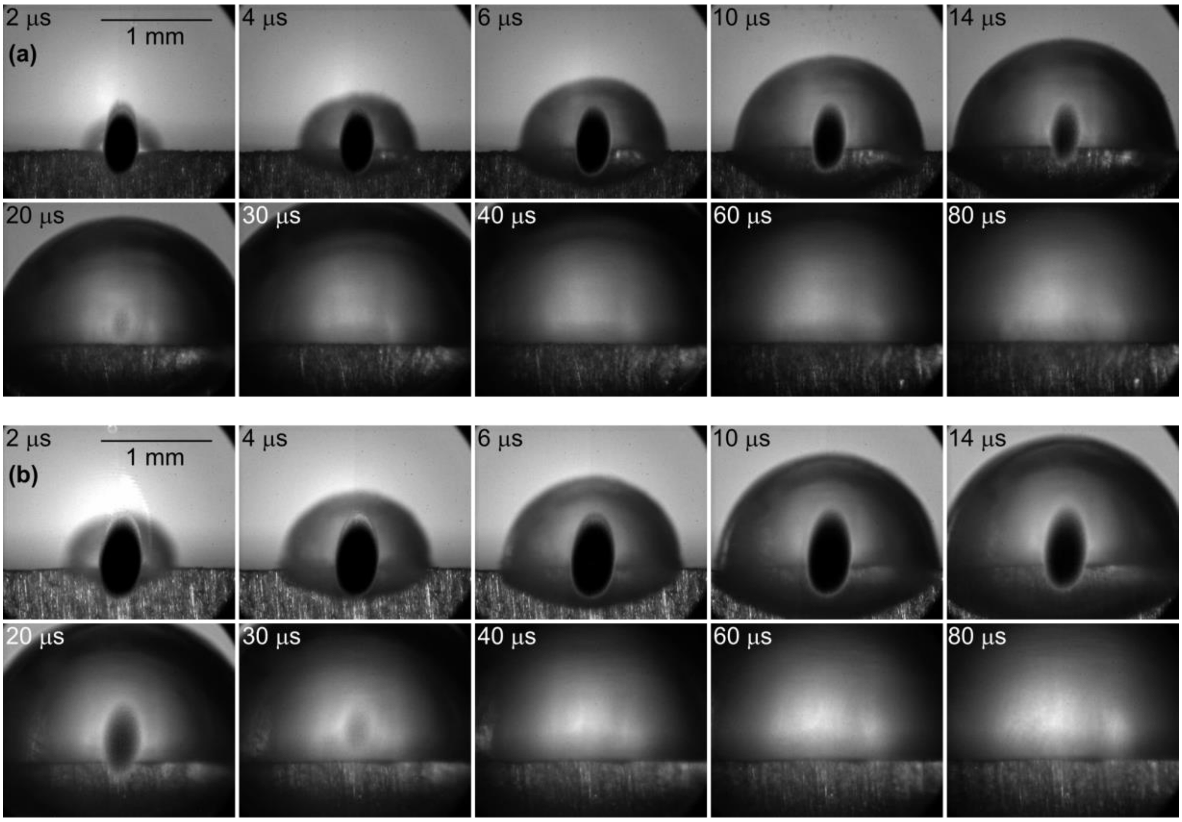

Fig. S31. Bubble dynamics of bubbles induced in (a) water (l = 0.2 mm) and (b) ethanol (l = 0.3 mm). Laser pulse energy equals 25 mJ.

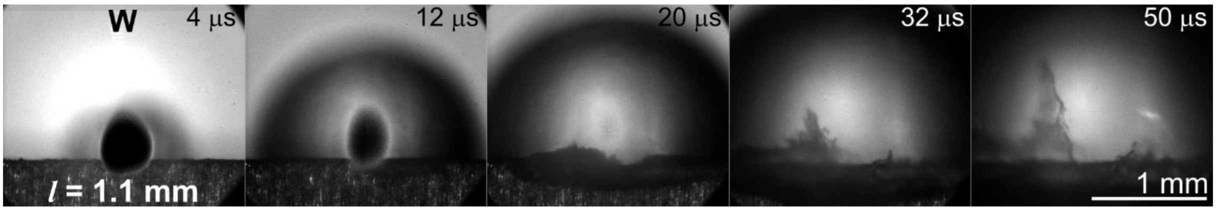

Fig. S32. Asymmetrical re-entrant injection dynamics in a bubble at l = 1.1 mm in water. Laser pulse energy equals 25 mJ.

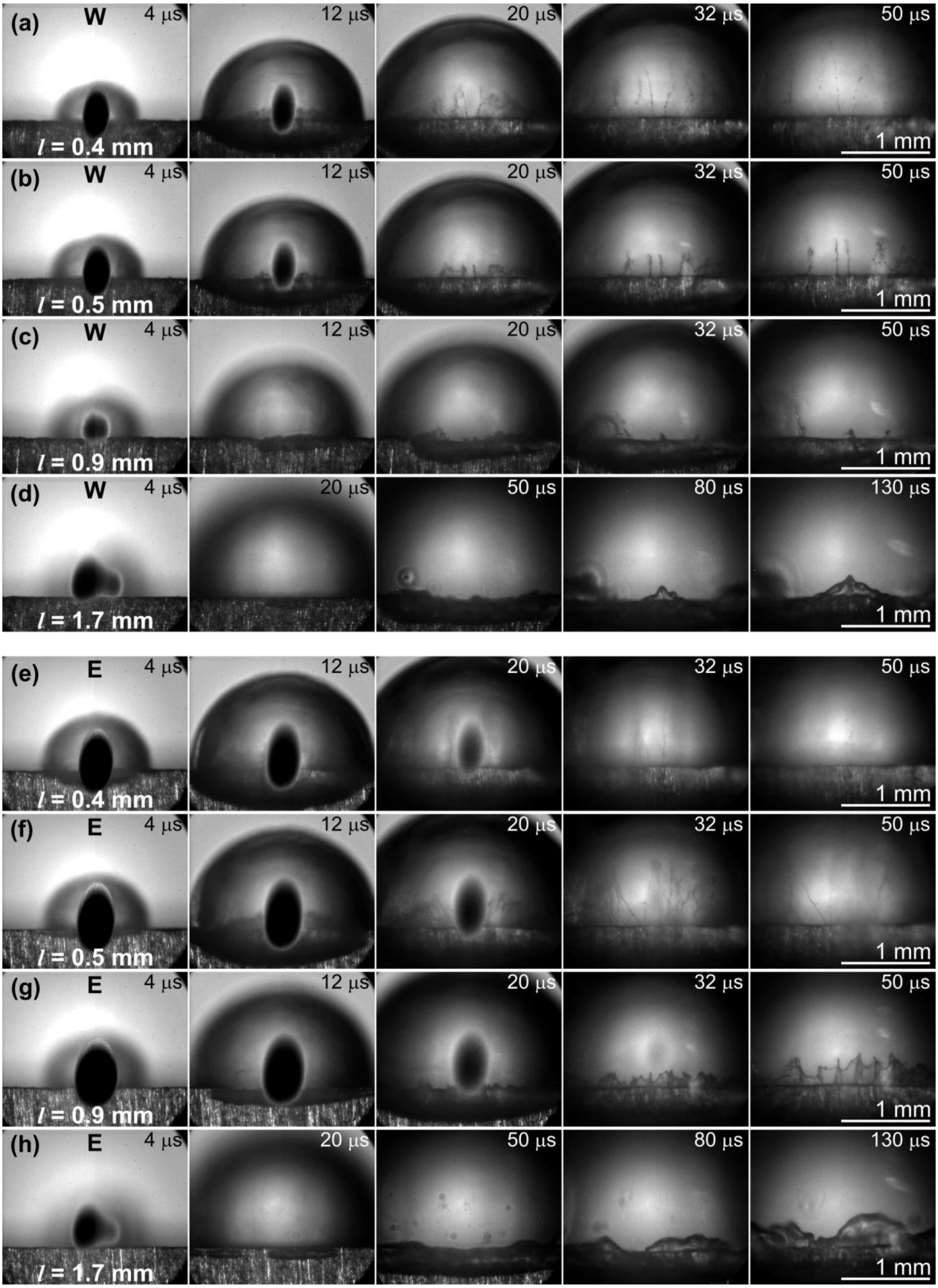

Fig. S33. Re-entrant injection dynamics in bubbles induced at l = 0.4 mm – 1.7 mm in (a-d) water and (e-h) ethanol. Laser pulse energy equals 25 mJ. Note the different time scale of (d) and (h) compared to others.

S5.4 Influence of pulse energy

Figures S34 and S35 show the influence of changing solely the pulse energy. As can be seen from Fig. S34 (l = 0.3 mm), increasing the pulse energy increases the jet velocity v_j and promotes more significant dispersion of the injection into smaller droplets (Fig. S34b, 40 μs). Similar effect is observed when decreasing the breakdown-edge distance at constant pulse energy. Decreasing the pulse energy, on the other hand, also decreases the jet velocity (Fig. S35a). The latter is also observed when l is increased at constant pulse energy.

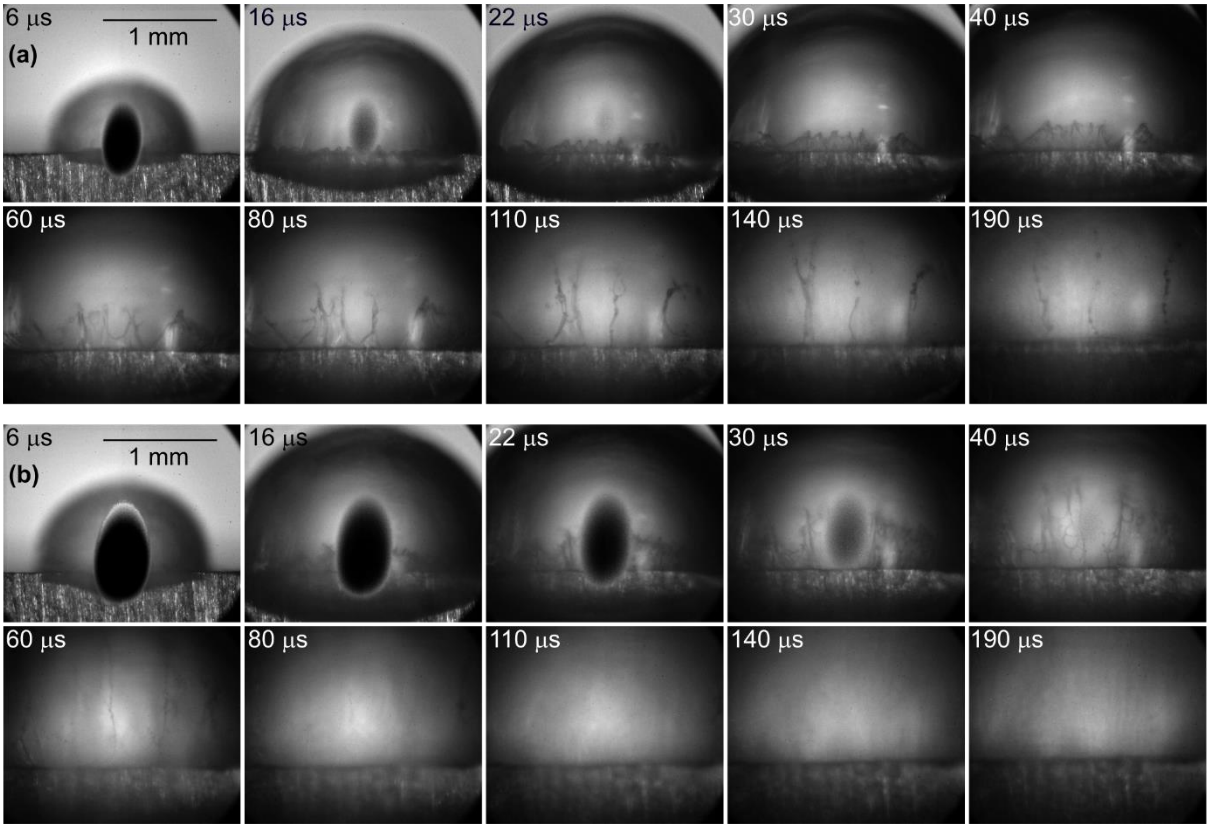

Fig. S34. Comparison of liquid injections in ethanol induced by laser pulses with pulse energies of (a) 10 mJ and (b) 25 mJ. Distance between the breakdown and the edge equals 0.3 mm.

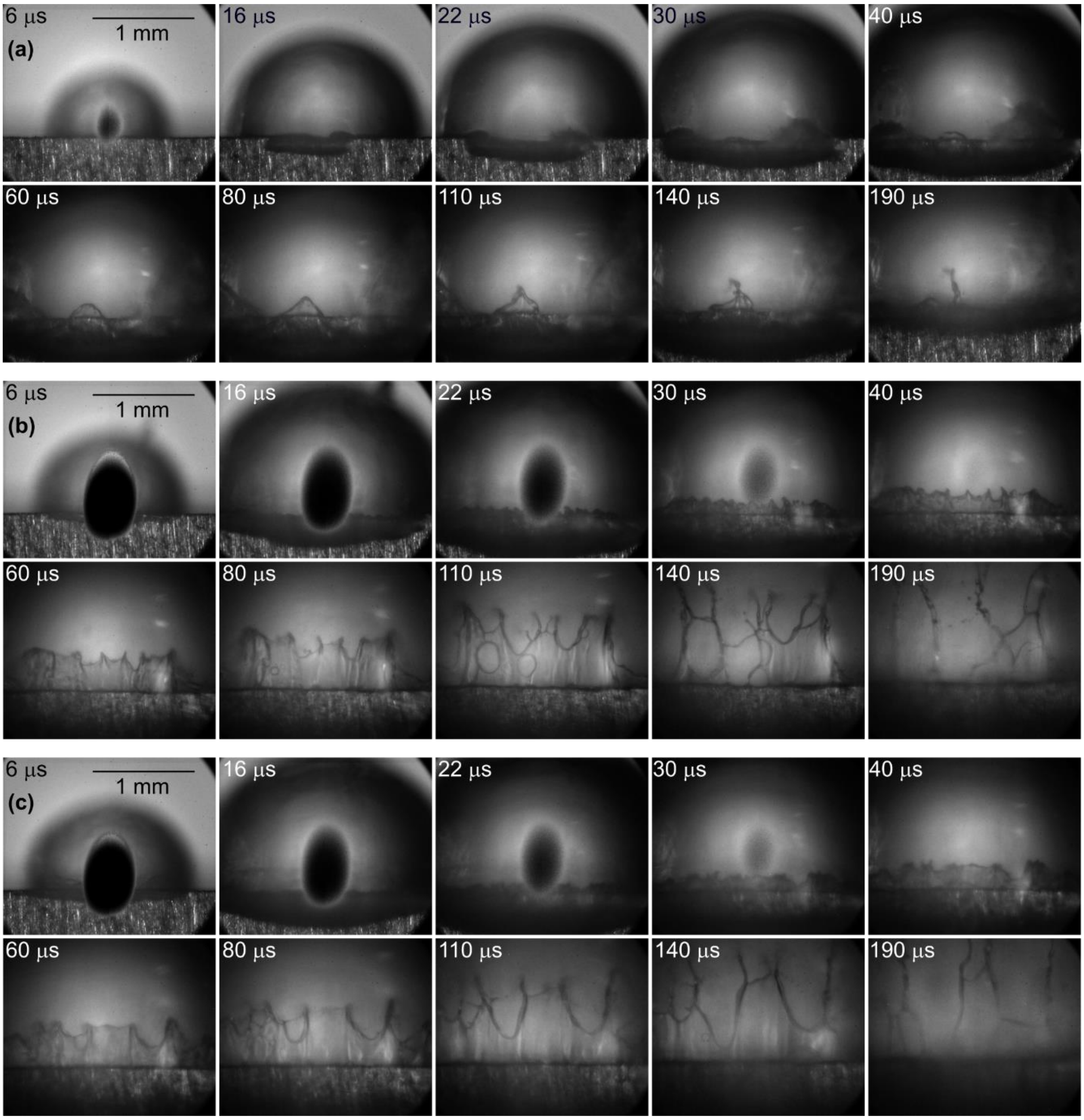

Fig. S35. Comparison of liquid injections in ethanol for pulse energies of (a) 10 mJ, (b) 25 mJ, and (c) 55 mJ. The breakdown-edge distance equals 0.9 mm.

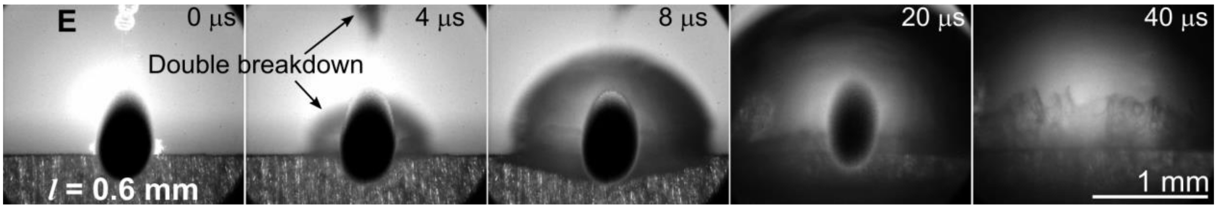

Fig. S36. Double breakdown in ethanol at pulse energy of 55 mJ and l = 0.6 mm.

S6 Influence of sample thickness

The effective distance between the position of breakdown and the edge was also varied by changing the thickness of the sample, denoted by d in Fig. S1. In this case, we clamped the sample in an "H" configuration (Fig. S1a) and the breakdown was induced in the middle of the sample, meaning $l = a = d/2$ according to notation in Fig. S1a.

The energy of the cavitation bubble in Fig. S37a is significantly lower due to smaller sample width compared to the laser spot size. Similarly to the already presented results of the influence of the breakdown-edge distance, the jet only appears when this distance is in the order of a few hundred micrometers, as seen in Fig. S37c. However, in this case, the "threshold" thickness of the sample, that is required for formation of liquid injection into the cavitation bubble, was not determined with good accuracy since we had no foil samples with thicknesses between 0.38 mm and 1 mm. Nevertheless, we presume that the mechanism of the jet formation is similar to that with the breakdown not induced in the middle of the sample, concluding that the jets at these laser parameters should occur at sample thicknesses excessing ~0.6 mm (thickness equal to the shortest breakdown-edge distance of the detected injections, multiplied by two).

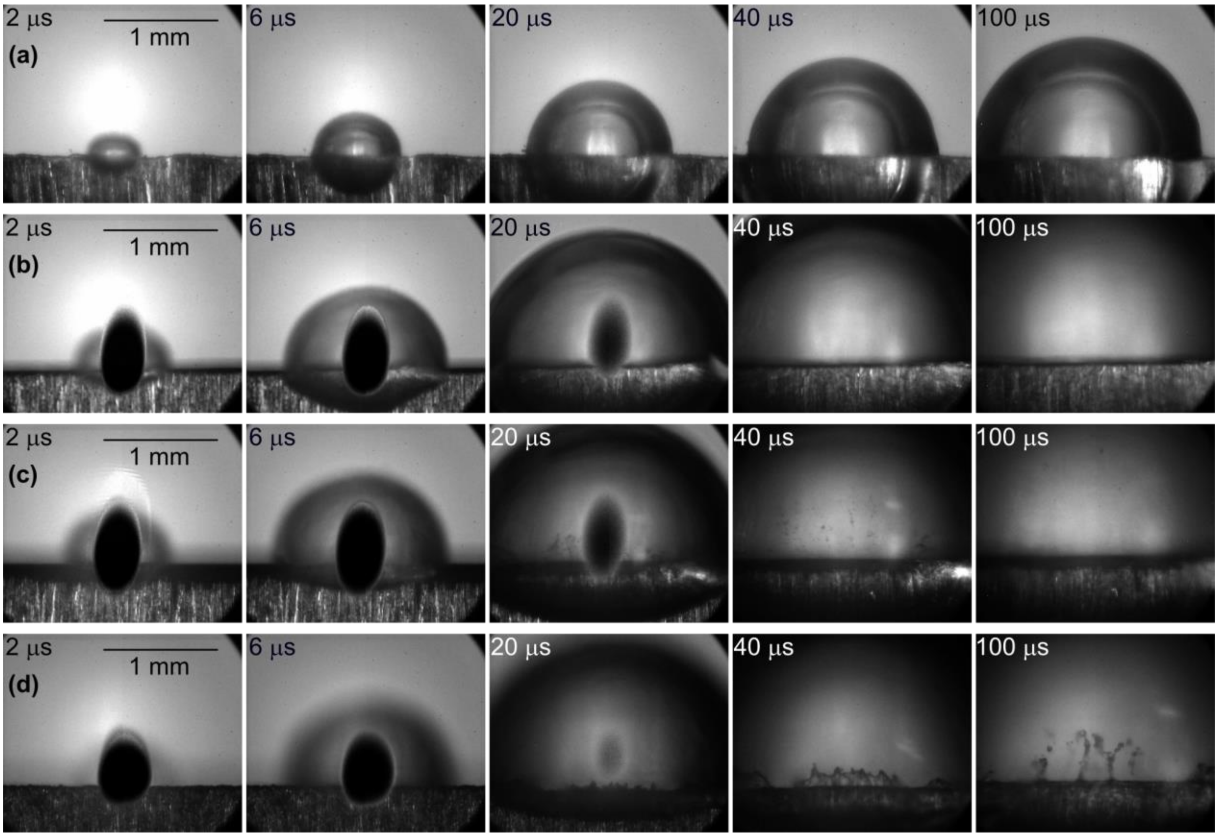

Fig. S37. Comparison of the bubble dynamics in water at different thicknesses of the samples. Pulse energy equals 25 mJ. Breakdown is induced in the middle of the sample with thickness of (a) 25 μm, (b) 0.38 mm, (c) 1 mm, and (d) 2 mm.

References

[S1] M. Born, E. Wolf, Principles of optics: electromagnetic theory of propagation, interference and diffraction of light, Elsevier, 2013.

[S2] C. Bongiovanni, A. Dominguez, J.-P. Chevaillier, Understanding images of bubbles, European Journal of Physics, 21 (2000) 561-570. https://doi.org/10.1088/0143-0807/21/6/307

[S3] D.L. Jones, Intermediate Strength Blast Wave, The Physics of Fluids, 11 (1968) 1664-1667. https://doi.org/10.1063/1.1692177.
